# Clinical Correlates of Compliance, Appeasement and Resistance in Command Hallucinations: A Systematic Review

**DOI:** 10.1002/cpp.70246

**Published:** 2026-03-14

**Authors:** M. Medas, A. Georgiades

**Affiliations:** ^1^ Department of Psychosis Studies, Institute of Psychiatry, Psychology, and Neuroscience (IoPPN) King's College London London UK; ^2^ Brent Early Intervention Service CNWL NHS Foundation Trust London UK

**Keywords:** appeasement, command hallucinations, compliance, psychosis, resistance, schizophrenia

## Abstract

**Objective:**

Command hallucinations (CHs) are a subtype of auditory hallucination commonly observed in psychosis and are strongly associated with harmful behaviours towards the self and others. Despite their clinical relevance, no review has synthesised the clinical variables associated with compliance, appeasement and resistance.

**Method:**

A systematic review was conducted to synthesise the existing evidence regarding the clinical correlates of compliance, appeasement and resistance to CHs.

**Results:**

Fifty‐six studies were eligible for inclusion. Compliance was associated with *cognitive factors* (benevolence, omnipotence and omniscience beliefs, perceived consequences of disobedience, perceptions of future compliance and greater attentive awareness), *relational factors* (social rank, voice identity and voice familiarity), *emotional drivers* (anger and obligation), *behaviours* (impulsivity and social isolation), *childhood trauma*, *substance use* and *overall symptom severity*. Appeasement was associated with *cognitive factors* (perceived dangerousness) and *behaviours* (avoiding provocation, obeying milder commands or self‐harm to protect others). Resistance was associated with *cognitive factors* (malevolence and omnipotence beliefs, perceived control over the voice and concurrent suicidal ideation) and *voice topography factors* (high intrusiveness/frequency/volume and low authoritativeness), alongside *childhood trauma factors* (interpersonal adversities and fearful attachment).

**Conclusion:**

These findings highlight the need for clinical formulations of CHs to attend closely to the factors driving compliance and appeasement, given their strong association with risk. Targeting the cognitive, relational, emotional, behavioural and developmental influences that sustain these responses—and strengthening resistance‐promoting factors—may enhance cognitive behavioural therapy for psychosis (CBTp). To support clinical practice, this review also provides Socratic questions to guide the assessment, formulation and intervention of CHs.

## Summary

- *Compliance* is associated with cognitive, relational, emotional, behavioural, developmental and substance‐related factors, as well as overall symptom severity.
- *Appeasement* is associated with cognitive and behavioural factors and represents a risk profile comparable to that of full compliance, underscoring its importance in routine clinical assessment.
- *Resistance* is associated with cognitive factors, voice topography and childhood trauma.
- Interventions targeting modifiable clinical correlates of compliance and appeasement (e.g., cognitive, relational, emotional, behavioural and substance‐related factors), alongside strengthening modifiable resistance factors (e.g., cognitive factors), may help reduce risk.
- The *Cognitive Model of Compliance, Appeasement, and Resistance to Command Hallucinations* enables the development of longitudinal and idiosyncratic case formulations regarding command hallucinations, which would enhance current CBTp practices.

## 1 Introduction

Command hallucinations (CHs) are a subtype of auditory hallucination commonly experienced in individuals with psychosis (Shawyer et al. 2003). They are characterised by voices that instruct an individual to perform specific actions (Hersh and Borum 1998) and have a median prevalence rate of approximately 53% among adult psychiatric patients with auditory hallucinations (range: 18%–89%) (Shawyer et al. 2003). Responses to CHs fall into three distinct categories: compliance, appeasement and resistance. *Compliance* involves fully carrying out the command as instructed by the voice (Braham et al. 2004). *Appeasement* refers to partial or negotiated compliance intended to minimise the perceived threat posed by the voice (Birchwood et al. 2014). In contrast, *resistance* involves actively opposing or refusing to enact a command (Chadwick and Birchwood 1994; Strauss et al. 2018). Although appeasement may appear less threatening than full compliance, partial acts of self‐harm (e.g., cutting or burning) can still lead to detrimental outcomes. These behaviours contrast more overtly dangerous acts (e.g., jumping from a window, running into traffic) but nevertheless remain of clinical importance. In individuals with CHs, 48% were found to engage in full compliance, 31% in appeasement/partial compliance and 21% in resistance behaviours (Shawyer et al. 2008). Harm‐other and self‐harm commands were reported by 48% and 44% of participants, respectively (Shawyer et al. 2008). CHs are thus considered harmful because they involve dangerous acts (Shawyer et al. 2003) and are indeed associated with suicidal behaviours and distress (Bucci et al. 2013; Wong et al. 2013).

Understanding the clinical correlates of compliance, appeasement and resistance can enhance risk assessment and inform targeted interventions. By focusing on reducing compliance and appeasement behaviours while promoting resistance, clinicians can more effectively manage risk and alleviate distress. Clinical variables associated with compliance include beliefs about the voice as omnipotent (Bucci et al. 2013) and appraising the voice intent as benevolent (Beck‐Sander et al. 1997). Appeasement has been associated with the perceived dangerousness of the voice and whether instructions are directed at others as opposed to oneself (Barrowcliff and Haddock 2010). Moreover, resistance has been associated with appraisals of the voice as malevolent (Chadwick, Lees, and Birchwood 2000; Chadwick et al. 2007). No study to date has comprehensively synthesised the clinical variables associated with each of these three dimensions of compliance, appeasement and resistance in CHs. This systematic review therefore aims to address this gap, with the view to informing clinical formulation, improving risk management strategies and supporting the development of targeted interventions in cognitive behavioural therapy for psychosis (CBTp). To ensure clarity, definitions for key CH‐related terms used throughout this review are provided in Table 1.

**TABLE 1:** Definitions for command hallucination terms.

Command hallucination term	Definition
Command hallucinations	Auditory hallucinations in which a person perceives voices giving directives, instructions or imperatives, ranging from benign actions to harmful or dangerous behaviours.
Compliance	Active adherence to the voice's instructions, involving engagement in the commanded behaviour. Influenced by perceived threat, authority of the voice or difficulty distinguishing hallucination from reality.
Appeasement	Partial compliance aimed at satisfying, placating or reducing perceived threat from the voice without fully following the command. Examples: symbolic actions, complying with non‐violent instructions, mentally rehearsing self‐harm or verbal gestures.
Resistance	Active refusal or inhibition of the voice's commands, including mental, emotional or behavioural strategies (e.g., ignoring, challenging, distraction, seeking support). Influenced by insight, perceived consequences, coping skills and voice relationship.
Power	Perceived influence, authority or control the voice holds over the individual, affecting compliance, resistance and distress.
Identity	Perceived source, personality or character of the voice (e.g., known person, supernatural being, authority figure), influencing interpretation and perceived threat.
Purpose	Perceived motivation or intention behind the voice (e.g., benevolent, malicious, protective, punitive, directive), shaping emotional responses and behavioural patterns.
Omnipotence	Belief that the voice has unlimited power or control over events, outcomes or the individual, which can increase anxiety and compliance.
Omniscience	Belief that the voice has complete knowledge of the individual's thoughts, intentions or past/future, potentially reducing resistance.
Social rank	Perceived hierarchical position relative to the voice (inferior, subordinate, equal, superior), influencing power dynamics, emotional responses and behaviours.
Benevolence	Perception that the voice has kind, protective or helpful intentions, which may reduce distress and promote collaboration.
Malevolence	Perception that the voice has harmful, threatening or punitive intentions, associated with distress and higher likelihood of appeasement or resistance.
Voice familiarity	Extent to which the voice is recognised or perceived as known, influencing emotional responses, credibility, authority and behavioural patterns.
Voice content	Specific messages, themes or instructions conveyed by the voice, including commands, commentary, threats or guidance, affecting emotional and behavioural responses.
Voice topography	Perceptual/spatial characteristics of the voice (e.g., location, lateralisation, volume, clarity), affecting perceived realism, intrusiveness and coping strategies.
Voice intrusiveness	Extent to which the voice disrupts thoughts, attention or functioning; highly intrusive voices cause distress and cognitive interference.
Voice authoritativeness	Perceived credibility or dominance of the voice, which can increase compliance, anxiety or obligation to obey.
Perceived control over commands	Subjective sense of agency over following, resisting or modifying the voice's directives; higher control reduces distress and increases resistance.
Hallucination‐related delusions	Fixed, false beliefs arising in response to hallucinations (e.g., beliefs about voice origin, power or intent), which can reinforce compliance, appeasement or distress.

### 1.1 Aims

The aim of this systematic review was to synthesise the existing evidence base regarding the clinical variables associated with compliance, appeasement and/or resistance to CHs in individuals with psychosis, with a view to supporting formulation development in CBTp.

## 2 Method

The PRISMA guidelines (Page et al. 2021) were followed, and this study was registered with the PROSPERO Register (registration number: CRD420251066871).

### 2.1 Search Strategy

A systematic search of MEDLINE (including PubMed), EMBASE, Global Health and APA PsycINFO was conducted using OVID from database inception to 30 June 2025. The following search strings were used: Psychosis OR Psychotic OR Schizophreni* **AND** Command hallucination* OR Auditory hallucination* OR Voice* **AND** Complian* OR Partial* complian* OR Comply OR Resist* OR Appeas* OR Engag* OR Respons* OR Relationship.

### 2.2 Study Selection

**FIGURE 1:**
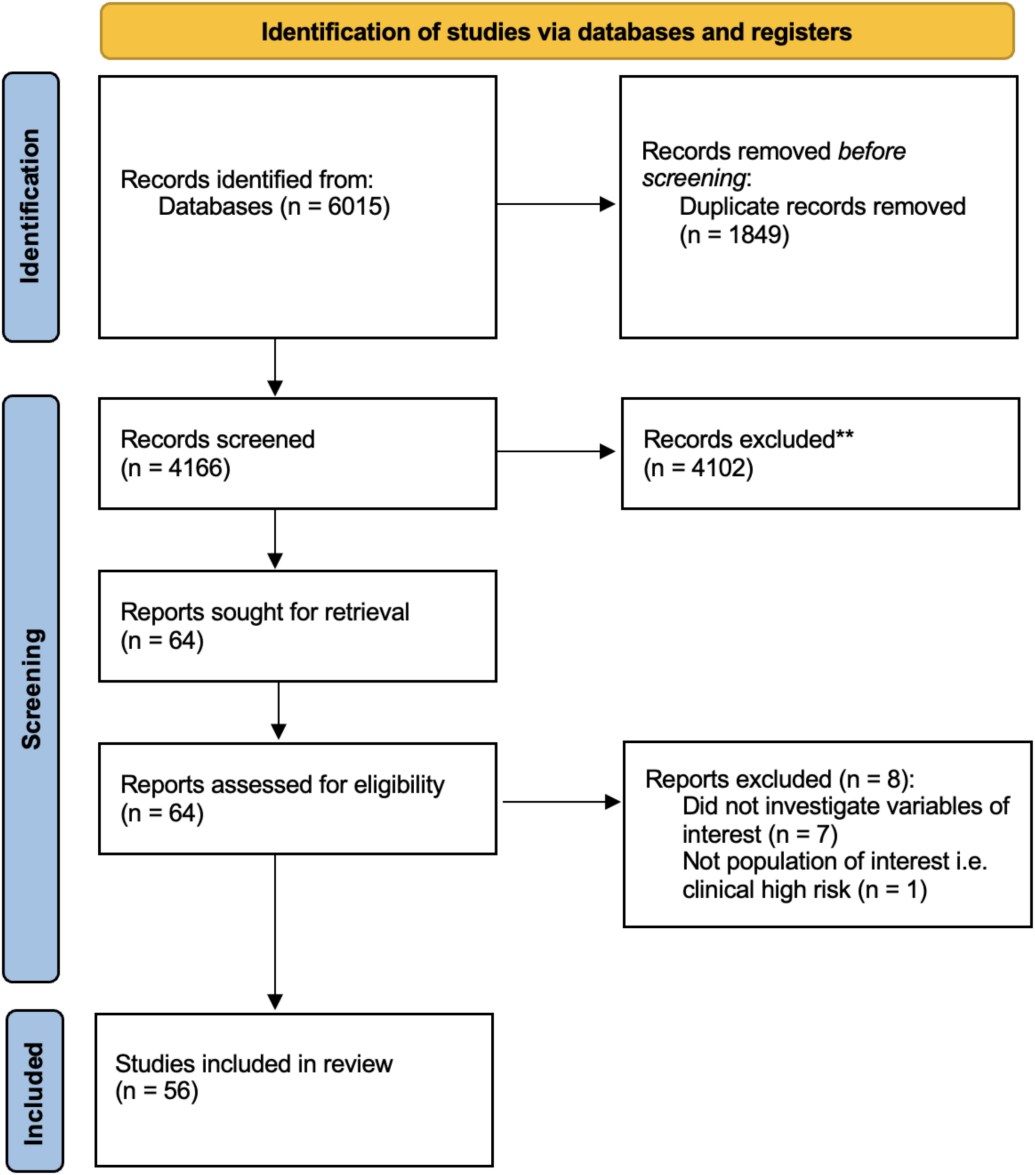
PRISMA 2020 flow diagram.

All study designs, regardless of their publication date or duration, were considered. Eligible studies needed to investigate the clinical factors associated with compliance, appeasement and/or resistance to CHs. Relevant studies were written in English, published in peer‐reviewed journals and included participants with a psychotic disorder diagnosed using a reliable psychometric tool (e.g., Diagnostic and Statistical Manual of Mental Disorders, fifth edition, or DSM‐5; American Psychiatric Association 2013; International Classification of Diseases, 11th edition, or ICD‐11; World Health Organisation 2019). The selection process is presented in the PRISMA flow diagram (see Figure 1).

### 2.3 Exclusion Criteria

Studies written in languages other than English, unpublished/grey literature, conference abstracts, book chapters, meta‐analyses, systematic reviews and case studies were excluded from this review. Studies that included samples with drug‐induced psychosis or psychosis due to organic causes only were also excluded.

### 2.4 Data Extraction Process

An Excel spreadsheet was created to record the characteristics of the included studies: author (name/year), country and type of study, sample size and setting, mean age (SD), mean duration of illness (SD), questionnaires/diagnostic tools, main findings and clinical implications. The screening process was conducted independently by one reviewer (M.M.) and was subsequently cross‐checked by a second reviewer (A.G.).

### 2.5 Quality Appraisal

The Effective Public Health Practice Project (EPHPP) (Thomas 2003) was used to assess the quality of quantitative studies (see Table 2), and the Joanna Briggs Institute Critical Appraisal Checklist for Qualitative Research (Joanna Briggs Institute; Lockwood et al. 2015) was used for qualitative studies (see Table 3).

**TABLE 2:** Quality assessment for quantitative studies—EPHPP tool.

Author (year)	Selection bias	Study design	Confounders	Data collection methods	Withdrawals and dropouts	Analyses (appropriateness)	Global rating
Andrew et al. (2008)	Strong	Moderate	Moderate	Strong	Strong	Yes	Strong
Barrowcliff and Haddock (2010)	Strong	Moderate	Moderate	Strong	Strong	Yes	Moderate
Beck‐Sander et al. (1997)	Strong	Moderate	Moderate	Moderate	Strong	Yes	Moderate
Begemann et al. (2022)	Strong	Moderate	Moderate	Strong	Strong	Yes	Strong
Birchwood and Chadwick (1997)	Strong	Moderate	Moderate	Strong	Strong	Yes	Strong
Birchwood and Trower (2006)	Weak	Strong	Moderate	Strong	Strong	Yes	Moderate
Birchwood et al. (2004)	Strong	Moderate	Weak	Strong	Strong	Yes	Moderate
Birchwood et al. (2018)	Strong	Strong	Moderate	Strong	Strong	Yes	Strong
Bucci et al. (2013)	Strong	Moderate	Moderate	Moderate	Strong	Yes	Strong
Chadwick and Birchwood (1995)	Strong	Moderate	Weak	Moderate	Strong	Yes	Moderate
Chadwick, Lees, and Birchwood (2000)	Moderate	Moderate	Weak	Strong	Weak	Yes	Weak
Chadwick et al. (2007)	Strong	Moderate	Weak	Strong	Weak	Yes	Weak
Chaix et al. (2014)	Strong	Moderate	Moderate	Strong	Strong	Yes	Strong
Chawla et al. (2019)	Strong	Moderate	Moderate	Strong	Strong	Yes	Strong
Close and Garety (1998)	Strong	Moderate	Moderate	Weak	Strong	Yes	Moderate
Dugré and West (2019)	Strong	Moderate	Moderate	Moderate	Strong	Yes	Strong
Dugré et al. (2018)	Strong	Moderate	Moderate	Moderate	Strong	Yes	Strong
Ellett et al. (2017)	Strong	Moderate	Moderate	Strong	Weak	Yes	Moderate
Erkwoh et al. (2002)	Strong	Moderate	Moderate	Weak	Strong	Yes	Moderate
Favrod et al. (2004)	Strong	Moderate	Moderate	Strong	Strong	Yes	Strong
Fielding‐Smith et al. (2022)	Strong	Moderate	Moderate	Strong	Strong	Yes	Strong
Fox et al. (2004)	Strong	Moderate	Moderate	Moderate	Strong	Yes	Strong
Ghadban et al. (2024)	Strong	Moderate	Moderate	Strong	Strong	Yes	Strong
Gmeiner et al. (2020)	Strong	Moderate	Moderate	Moderate	Strong	Yes	Strong
Hacker et al. (2008)	Strong	Moderate	Moderate	Moderate	Strong	Yes	Strong
Hazell et al. (2024)	Strong	Moderate	Moderate	Moderate	Strong	Yes	Strong
Junginger (1990)	Strong	Moderate	Weak	Weak	Strong	Yes	Weak
Junginger (1995)	Strong	Moderate	Moderate	Weak	Strong	Yes	Moderate
Lee et al. (2004)	Strong	Moderate	Strong	Weak	Strong	Yes	Moderate
Lucas and Wade (2001)	Strong	Moderate	Moderate	Strong	Strong	Yes	Strong
Mackinnon et al. (2004)	Strong	Moderate	Moderate	Moderate	Strong	Yes	Strong
Marotti et al. (2025)	Strong	Strong	Strong	Moderate	Strong	Yes	Strong
Morris et al. (2014)	Strong	Moderate	Moderate	Strong	Weak	Yes	Moderate
Peters et al. (2012)	Strong	Moderate	Moderate	Strong	Strong	Yes	Strong
Rajanthiran et al. (2022)	Strong	Moderate	Moderate	Strong	Strong	Yes	Strong
Reynolds and Scragg (2010)	Weak	Moderate	Moderate	Strong	Moderate	Yes	Moderate
Robles‐García et al. (2004)	Strong	Moderate	Moderate	Strong	Strong	Yes	Strong
Rogers et al. (2002)	Moderate	Moderate	Moderate	Moderate	Strong	Yes	Strong
Salim et al. (2021)	Strong	Moderate	Moderate	Moderate	Strong	Yes	Strong
Sayer et al. (2000)	Strong	Moderate	Moderate	Strong	Moderate	Yes	Strong
Shawyer et al. (2007)	Strong	Moderate	Moderate	Moderate	Strong	Yes	Strong
Shawyer et al. (2008)	Strong	Moderate	Moderate	Moderate	Moderate	Yes	Strong
Simms et al. (2007)	Strong	Moderate	Moderate	Strong	Strong	Yes	Strong
So and Wong (2008)	Strong	Moderate	Moderate	Moderate	Strong	Yes	Strong
So et al. (2016)	Moderate	Moderate	Strong	Strong	Strong	Yes	Strong
So et al. (2021)	Strong	Moderate	Moderate	Strong	Strong	Yes	Strong
Soppitt and Birchwood (1997)	Weak	Moderate	Moderate	Moderate	Strong	Yes	Moderate
Stephanie et al. (2018)	Moderate	Moderate	Moderate	Strong	Strong	Yes	Strong
Trower et al. (2004)	Strong	Moderate	Moderate	Moderate	Moderate	Yes	Moderate
van der Gaag et al. (2003)	Strong	Moderate	Moderate	Strong	Moderate	Yes	Strong
Zanello and Dugré (2021)	Strong	Moderate	Moderate	Moderate	Strong	Yes	Strong

Abbreviation: EPHPP, Effective Public Health Practice Project (Thomas 2003).

**TABLE 3:** Quality assessment for qualitative studies—JBI tool.

Author (year)	1. Is there congruity between the stated philosophical perspective and the research methodology?	2. Is there congruity between the research methodology and the research question or objectives?	3. Is there congruity between the research methodology and the methods used to collect data?	4. Is there congruity between the research methodology and the representation and analysis of data?	5. Is there congruity between the research methodology and the interpretation of results?	8. Are participants, and their voices, adequately represented?	9. Is the research ethical according to current criteria or, for recent studies, and is there evidence of ethical approval by an appropriate body?	10. Do the conclusions drawn in the research report flow from the analysis, or interpretation, of the data?	Include if yes to 2–5, 8–10
Chadwick and Birchwood (1994)	Yes	Yes	Yes	Yes	Yes	Yes	Unclear	Yes	✓
Denno et al. (2022)	Yes	Yes	Yes	Yes	Yes	Yes	Yes	Yes	✓✓
Fenekou and Georgaca (2009)	Yes	Yes	Yes	Yes	Yes	Yes	Unclear	Yes	✓
Kalhovde et al. (2014)	Yes	Yes	Yes	Yes	Yes	Yes	Yes	Yes	✓✓
Suryani et al. (2013)	Yes	Yes	Yes	Yes	Yes	Yes	Yes	Yes	✓✓

Abbreviation: JBI, Joanna Briggs Institute Quality Assessment Tool (Lockwood et al. 2015).

## 3 Results

Of the 6015 studies initially identified during screening, a total of 56 papers met inclusion. The characteristics, main findings and clinical implications for the eligible studies are described in Table 4.

**TABLE 4:** Characteristics of studies meeting inclusion criteria (*n* = 56).

Author (year)	Country and type of study	Sample size and setting	Mean age (SD) and mean duration of illness (SD)	Questionnaire and diagnostic tools	Main findings and clinical implications
Andrew et al. (2008)	UK Cross‐sectional	22 psychiatric voice hearers (13M/9F) 21 non‐psychiatric voice hearers (6M/15F) Outpatient	Mean age of psychiatric voice hearers 39.55 (12.3) Mean length of time hearing voices in psychiatric voice hearers 12.91 (10.1) Mean age of non‐psychiatric voice hearers 50.67 (11.3) Mean length of time hearing voices in non‐psychiatric voice hearers 30.62 (15.77)	Belief About Voices Questionnaire–Revised (BAVQ‐R) Impact of Events Scale (IES) Post‐traumatic Diagnostic Scale (PDS)	Psychiatric Voice Hearers (PVH) believed the voices to be significantly more malevolent and omnipotent than Non‐Psychiatric Voice Hearers (NPVH). PVH also reported significantly more resistant coping behaviours. Trauma variables comprising of re‐experiencing, avoidance and arousal, significantly predicted beliefs regarding malevolence and omnipotence, accounting for 66.4%, 48.5% and 42.5% of variance in beliefs respectively. The total score on the IES was the only significant predictor of malevolence, benevolence and omnipotence. These results indicate that current symptoms related to past traumatic events are significant predictors of a person's beliefs about their voices, with greater current trauma symptoms being associated with more malevolence and omnipotence (and less benevolence) of the voice.
Barrowcliff and Haddock (2010)	UK Cross‐sectional	49 SZ or schizoaffective disorder with command hallucinations (29M/20F) Inpatient and outpatient	Mean age 36.7 (11.8) Mean time since diagnosis 13.2 (10.7)	Beliefs about Voices Questionnaire (BAVQ) Gudjonsson Compliance Scale (GCS) Positive and Negative Syndrome Scale (PANSS) Social Comparison Scale (SCS)	This study found that compliance with the last self‐harm command was associated with elevated voice malevolence, heightened symptom presentation and perceived consequences for non‐compliance. Compliance with the last harm‐other command was associated with elevated symptom severity, higher perceived consequences for non‐compliance and higher levels of voice social rank. However, these associations were not maintained for compliance during the previous 28 days. Higher rates of partial compliance for commands directing harm at others compared to self‐harm and an absence of partial compliance in regard to benign commands were observed. Depending upon the type of commands experienced as distressing by the client, elements of therapeutic focus should target the components most associated with the likelihood of compliance, to reduce subsequent risk for the client and those who may be implicated in the hallucinatory experience. These authors suggest that due to the high levels of risk of harm to self and others every effort must be made to further understand and help manage such experiences within such clinical cohorts.
Beck‐Sander et al. (1997)	UK Cross‐sectional	35 SZ or related disorder who experienced command hallucinations (25M/10F) Inpatient rehabilitation service	60% of participants 25–35 34% > 35 6% < 25 All participants had experienced hallucinations for at least 1 year	Beliefs About Voices Questionnaire (BAVQ) Semi‐structured interview	This study found that a belief that the voice is benevolent was associated with compliance with both innocuous and severe commands. In addition, participants who believed they retained subjective control over their voices were less likely to comply with all types of command. There is also evidence that participants were more willing to comply with commands to harm themselves than they were to comply with commands to hurt others. Acts of appeasement often involved incidents of self‐harm. Participant 3 believed he heard the voice of the devil telling him to attack a member of staff and he slashed his own wrists hoping that would satisfy the voice. Participant 15 experienced a voice telling him to forcibly perform oral sex on a female patient against her will. He says he was aroused but also felt guilty. He attempted to appease the voices by swallowing varnish and fishing shot.
Begemann et al. (2022)	The Netherlands Cross‐sectional	299 low trauma cluster (118M/181F) 71 emotion‐focused trauma cluster (16M/55F) 43 multi‐trauma cluster (22M/21F) Outpatient	Mean age low trauma cluster 40.01 (13.55) Mean age emotion‐focused trauma cluster 43.48 (11.38) Mean age multi‐trauma cluster 41.99 (13.49) Mean age of onset low trauma cluster 20.20 (14.28) Mean age of onset emotion‐focused trauma cluster 12.20 (12.81) Mean age of onset multi‐trauma cluster 15.33 (12.23)	Beliefs About Voices Questionnaire (BAVQ‐R) Childhood Trauma Questionnaire–Short form (CTQ‐SF)	This study found that the multi‐trauma cluster rated their voices as more malevolent and felt more resistance against them, compared to the low trauma cluster and the emotion focused trauma cluster. Furthermore, AVH were rated as significantly more omnipotent by the multi‐trauma compared to the low trauma cluster. Clinical implications include the need for tailored treatment approaches based on the different trauma subtypes and the potential pathways and mechanisms these reflect. For example, if some voices arise through posttraumatic stress reactions (e.g., intrusions and dissociation) to multiple trauma exposures, then this particular group should respond to trauma‐focused therapies (Brand et al. 2021). In comparison, those with low levels of trauma, but persisting voices may benefit more from traditional CBT for psychosis (Lincoln and Peters 2019). Such a theory could be tested by observing relative responses to different treatments in these groups.
Birchwood and Chadwick (1997)	UK Cross‐sectional	62 voice hearers with SZ or schizoaffective disorder (43M/19F) Outpatient	Mean age 39.0 (11.8) Mean duration of illness 16.5 (10.6)	Beliefs about Voices Questionnaire (BAVQ) Cognitive Assessment Schedule (CAS) Hustig and Hafner's (1990) voice topography scale The Psychiatric Assessment Scale (PAS)	This study found that malevolent voices were associated with fear and anger and were resisted, and benevolent voices were associated with positive effect and were engaged. Measures of voice form and topography did not show any link with behaviour or affect.
Birchwood and Trower (2006)	UK RCT	18 SZ or other related disorder receiving cognitive therapy for CHs (10M/8F) 20 SZ or other related disorder receiving TAU (14M/6F) Forensic	Mean age SZ or other related disorder receiving cognitive therapy for CHs 36.6 (10.3) Mean duration of voices SZ or other related disorder receiving cognitive therapy for CHs 13.4 (9.9) Mean age SZ or other related disorder receiving TAU 35.1 (10.4) Mean duration of voices SZ or other related disorder receiving TAU 10 (5.7)	Beliefs about Voices Questionnaire (BAVQ) Voice Power Differential Scale (VPD) Omniscience Scale (OS)	This study found that from 100% compliance they all significantly dropped, the TAU to 53% but the CBT group dropped significantly more, over 12 months to only 14% still complying or appeasing. If this very large reduction was due specifically to cognitive therapy, we would also expect to see a change in conviction in the power beliefs. In fact, that is what we did find. The CBT group reported a large and significant reduction in their beliefs in the power of the dominant voice, compared to TAU which showed no change, and this effect of CBT was maintained at 12‐month follow up. Furthermore, when we statistically removed the effect of the power beliefs, the treatment effect disappeared, providing further evidence that it was belief change that was responsible for the reduction in compliance. The belief in voices' omniscience also fell significantly in the CBT group but not in the TAU group, and this pattern was also maintained at 12 months. Patients receiving CBT also showed a significant improvement in perceived control over voices, compared to TAU, which showed no change. This pattern was maintained at 12 months. The data presented here suggests that CBT for CH, in the context of good quality and a high level of TAU services, exerts a major influence on the risk of compliance, reduces distress and prevents the escalation of depression, compared to TAU alone. Because of the selection criterion of recent compliance, it was likely that compliance behaviour would reduce over the 6‐ and 12‐month periods (‘regression to the mean’); however, given the high‐risk status of this group, we may expect an increasing number of people complying with commands as further time elapses. Nevertheless, the 12‐month clinical impact of CBT was significant. Perhaps more importantly, the risk factors for compliance in the CBT group had reduced markedly, particularly: the perceived power of the voice, its omniscience and controllability, and the need to appease (14% of the CBT group were appeasing or complying vs. 53% of TAU).
Birchwood et al. (2004)	UK Cross‐sectional	125 SZ or related disorder (85M/40F) Outpatient	Mean age 33.7 (9.3) Mean duration of illness not stated	Beliefs About Voices Questionnaire (BAVQ) Voice Power Differential scale (VPD)	This study found that voices rated as powerful and malevolent were closely linked to the ‘resistance’ coping strategy (*r* = 0.73, *p* < 0.01), whereas ‘benevolent’ voices were linked with engagement strategies (*r* = 0.60, *p* < 0.01). If malevolent voices are a form of (intense and often very nasty) bullying that may be rooted in earlier traumatic experiences and harassment (Birchwood 2003), then the therapist needs to align himself with the patient in reducing the experience of being bullied. Exploring possible shamed‐based origins of feeling subordinated (e.g., abuse) is important. Hence, cognitive therapists new to this area of work may need to be mindful of this kind of (bullying) experience, the power and fear of shame and thus extend their work beyond treating voices as intrusive thoughts or misattributions to which a person can be encouraged to apply cognitive behavioural techniques.
Birchwood et al. (2018)	UK RCT	197 SZ or related disorder with a history of command hallucinations (113M/84F) Outpatient	Mean age 37.4 (12.1) Mean duration of illness not stated	Beliefs about Voices Questionnaire‐Revised (BAVQ‐R) Calgary Depression Rating Scale for Schizophrenia Childhood Trauma Questionnaire (CTQ) Voice Compliance Scale (VCS) Voice Power Differential Scale (VPD‐total)	This study found that voice omnipotence was the best predictor (of compliance) although the principle component analysis identified a highly predictive cognitive‐affective dimension comprising voices' power, childhood trauma, depression and self‐harm. In the mediation analysis, the indirect effect of treatment was fully explained by its effect on the hypothesised mediator: voice power differential. The best predictor was BAVQ Omnipotence with stronger beliefs linked to compliance. This trial mediation analysis brings to full circle our influential cognitive model we formulated over 20 years ago (Chadwick and Birchwood 1994). We argue that it provides convincing evidence that in relation to harmful behaviour at least, the perceived power of voices to threaten the individual, relative to the perceived power of the individual to challenge and mitigate this threat (power differential), is a strong and malleable influence on voice‐related behaviour. Had the mediation analysis not supported the role of voice power, this would have questioned the foundation of the therapy in its focus on voice power differential and raised questions about other active agents, for example, demand characteristics of the trial, a placebo effect or bias. On the contrary, the mediation analysis supports the clinical model and opens the door for further research.
Bucci et al. (2013)	UK Cross‐sectional	32 command hallucination hearers; 16 SZ, 2 schizoaffective disorder 14 psychosis not otherwise specified (23M/9F) Inpatient and outpatient	Mean age 37.09 (11.36) Mean duration of illness not stated	Barrat Impulsiveness Scale (BIS‐11) Beliefs About Voices Questionnaire Revised (BAVQ‐R) Command Hallucinations Interview (CHI) Novaco Anger Scale and Provocation Inventory (NAS‐PI)	This study found that the tendency to appraise the voice as powerful, to be impulsive, to experience anger and to regulate anger were significantly associated with compliance with command hallucinations to do harm. Two factors emerged as significant independent predictors of compliance with command hallucinations: omnipotence and impulsivity. These findings provide preliminary support to suggest that the psychological factors associated with violence, aggression, suicide and self‐harm generally may be applicable to the same behaviours when they occur in the context of a response to a command hallucination. This has important clinical implications. Research on the factors associated with compliance with commands has led to the development of therapeutic interventions based on reducing the impact of compliance (Meaden et al. 2013). The current findings could indicate that, in addition to the cognitive approach to commands, aspects of anger and impulsiveness might also be worth consideration when assessing and formulating the risk of a person complying with command hallucinations, and when working therapeutically with these patients. Larger prospective studies examining the role of anger and impulsiveness in relation to compliance with specific command types, and the possible interaction effects between voice power and impulsiveness and their combined influence on compliance, are needed. The current data are highly suggestive and warrants replication with a larger sample.
Chadwick and Birchwood (1994)	UK Qualitative	26 SZ or schizoaffective disorder who had heard voices for at least 2 years (14M/12F) Inpatient and outpatient	Mean age 35 (range 23–59) Mean duration of illness not stated	Semi‐structured interviews	It was found that voices believed to be malevolent provoked fear and were resisted and those perceived as benevolent were courted. However, in the case of imperative voices, the primary influence on whether commands were obeyed was the severity of the command. The authors classified the commands as mild or severe (i.e., life‐threatening). Immediately one parameter can then be established. Fourteen voices (eight benevolent, six malevolent) gave only mild commands; all these benevolent voices were complied with willingly and in full, and all but one malevolent voice was complied with in full, although reluctantly—the one exception was S3. Severe commands were given by 12 voices (one benevolent, 11 malevolent) and all were currently being resisted. However, 10 of the 12 voices that gave severe commands also gave mild ones, and in all 10 cases these were obeyed at least occasionally. It is as if compliance with mild commands was an attempt to appease the voices' requirement for sterner actions. Of those people who were uncertain about the voice, two complied partially and one not at all. The present research strongly suggests that degree of distress is inextricably bound to subjective meaning, and that weakening critical beliefs about the voices might alleviate much of the associated distress and difficulty.
Chadwick and Birchwood (1995)	UK Cross‐sectional	60 SZ or schizoaffective disorder with chronic hallucinatory voices (42M/18F) Setting not stated	Mean age 39.9 (12.2) Mean duration of illness 16.3 (10.3)	Cognitive Assessment Schedule (CAS) Beliefs About Voices Questionnaire (BAVQ)	This study found a close relationship between malevolence and resistance on the one hand (*r* = 0.76) and benevolence and engagement on the other (*r* = 0.82); all other correlations were strongly negative. We therefore believe the Beliefs About Voices Questionnaire (BAVQ) to be a useful aid to assessment; also, the promising attempts to apply cognitive therapy to voices (Fowler and Morley 1989; Kingdon and Turkington 1991; Chadwick and Birchwood 1994; Haddock et al. 1993) suggest it should have useful treatment implications.
Chadwick, Lees, and Birchwood (2000)	UK Cross‐sectional	73 SZ or related disorder with drug‐resistant auditory hallucinations (41M/32F) Outpatient	Mean age 40 (10.91) Mean duration of illness not stated	Beliefs About Voices Questionnaire Revised (BAVQ‐R) Hospital Anxiety and Depression Scale (HADS)	This study found a strong relationship between malevolence and resistance (*r* = 0.68, *p* < 0.01) and benevolence and engagement (*r* = 0.80, *p* < 0.01), with all other correlations between these sub‐scales being strongly negative. There was also a relationship between depression and both omnipotence (*r* = 0.44, *p* < 0.01) and resistance (*r* = 0.32, *p* < 0.05). Depression was negatively associated with engagement (*r* = −0.42, *p* < 0.01). Participants continue to find the measure acceptable and easy to complete. The BAVQ‐R is a useful assessment and outcome measure. The measure gives a quick, reliable profile of a person's relationship with an auditory hallucination, which is useful information for psychological therapy.
Chadwick et al. (2007)	UK Cross‐sectional	59 SZ who experienced AHs (35M/24F) Inpatient and outpatient	Mean age 38.9 (11.9) Mean duration of illness 14.54 (11.71)	Beliefs About Voices Questionnaire Revised (BAVQ‐R) Southampton Mindfulness of Voices Questionnaire (SMVQ)	This study found significant negative correlations between the Southampton Mindfulness of Voices Questionnaire (SMVQ) and Malevolence (*r* = −0.50, *p* = 0.001, *n* = 58), Omnipotence (*r* = −0.65, *p* = 0.001, *n* = 58) and Resistance (*r* = −0.45, *p* = 0.001, *n* = 59).
Chaix et al. (2014)	Switzerland Cross‐sectional	28 with AVHs; 27 SZ 1 schizoaffective disorder (18M/10F) Outpatient	Mean age 36.5 (9.6) Mean duration of illness not stated	Beliefs About Voices Questionnaire (BAVQ) French version Safety‐seeking behaviours questionnaire (SBQ)	This study found that the Safety Behaviour Questionnaire (SBQ) (which measures safety behaviours such as avoidance, compliance, help‐seeking and confrontation) total score correlated significantly with the Omnipotence and resistance scales of the BAVQ. The significant predictive variables are beliefs about origin of voices resistance towards the voices and omnipotence of the voices. These results indicate that beliefs about origin of voices, voice resistance and omnipotence play an important role in predicting safety‐seeking behaviours. It would be useful in next cognitive behavioural therapy of verbal auditory hallucinations studies to examine how improvements in characteristics of voices and different cognitive variables can predict reduction of safety‐seeking behaviours.
Chawla et al. (2019)	India Cross‐sectional	30 SZ with AHs (19M/11F) Outpatient	Median age 32 (not stated) Mean duration of illness 7 (not stated)	Beliefs about Voices Questionnaire‐Revised (BAVQ‐R) Hindi version Clinical Global Impression‐Schizophrenia scale (CGI‐SCH) Psychotic Symptom Rating Scale (PSYRATS) Scale for the Assessment of Negative Symptoms (SANS) Scale for the Assessment of Positive Symptoms (SAPS)	This study found higher BAVQ‐R scores were found on malevolence, omnipotence and emotional and behavioural resistance. These beliefs had a significant positive correlation with the PSYRATS hallucination subscale, but not with the severity of psychosis SAPS, SANS and CGI‐SCH. The sample had lower scores for benevolence and engagement subscales of BAV‐Q. The findings have implications for clinical behavioural interventions which can be planned after a better understanding of the beliefs and experiences of treatment‐seeking patients who continue to have prominent AHs. Certain aspects can be focused on non‐pharmacological interventions for AHs. The therapist may gain some useful perspectives about beliefs for voices which can then be used for nonpharmacological therapies for persistent AH. For example, patients with higher engagement with the voices might benefit from distraction techniques. On the other hand, patients who show higher scores on malevolence/omnipotence/resistance may benefit from therapies teaching them better coping with the symptom, like adaptive CBT.
Close and Garety (1998)	UK Cross‐sectional	30 SZ with AHs (20M/10F) Outpatient	Mean age 40 (11.4) Mean duration of hearing voices 13 (9.1)	Cognitive Assessment of Voices (CAV)	This study found that participants reported that they were more likely to engage in any benevolent voices (63%) and resist malevolent voices (77%).
Denno et al. (2022)	UK Qualitative	35 psychosis and non‐psychosis diagnoses (gender unclear) Outpatient	Age range 17–37 Mean duration of illness not stated	Unstructured interviews	This study found other strategies used to cope with commanding voices included deliberately complying with or appeasing AVH (8/25 and 6/10 participants), avoiding provoking AVH, and avoiding situations in which losing control could be dangerous. Most reported being able to negotiate with or overcome voices some of the time, dependant on situation, mood and intensity. *Sometimes I can tell them no I do not want to do that, but like it's hard to not follow them 'cause it feels like if I follow them they'll get quieter (P123)* *It depends what mood I'm in. If I'm in a bad mood, they are in charge of me or sometimes I can, like, be in charge of them, like, tell them to go, or just get rid of them. (P124)* These results provide insight for researchers and clinicians. They highlight the complex and interactive process of identity‐formation occurring between voice and hearer and the influence of voice‐hearers' life‐history and belief‐system on this process.
Dugré and West (2019)	Canada Cross‐sectional	7 neutral beliefs group (4M/3F) 102 malevolent beliefs group (52M/50F) 14 benevolent beliefs group (10M/4F) 58 benevolent‐malevolent beliefs group (29M/29F) Inpatient	Mean age neutral beliefs group 27.1 (6.8) Mean years since first AVH neutral beliefs group 8.43 (9.7) Mean age malevolent beliefs group 29.3 (6.1) Mean years since first AVH malevolent beliefs group 7.92 (8.8) Mean age benevolent beliefs group 33.5 (5.85) Mean years since first AVH benevolent beliefs group 10 (10.6) Mean age benevolent‐malevolent beliefs group 30.9 (6.06) Mean years since first AVH 11.4 (9.7)	Auditory Hallucinations Schedule (AHS) Brief Psychiatric Rating Scale (BPRS‐18) Questionnaire about Childhood Experiences	This study found that participants with benevolent voices more frequently denied using coping strategies to manage voices and were more certain that they would obey in the future, than the malevolent and the benevolent‐malevolent (BM) groups. The multivariate model of the malevolent group revealed that frequency of childhood physical abuse (CPA) (OR = 1.59, 95% CI: 1.01–2.52, *p* = 0.047), feeling having to obey (OR = 1.42, 95% CI: 1.05–1.90, *p* = 0.021), conceptual disorganisation (OR = 2.23, 95% CI: 1.14–4.40, *p* = 0.020) and unusual thought content (OR = 1.28, 95% CI: 1.00–1.62, *p* = 0.046) significantly predicted, independently, frequency of lifetime compliance with CH.
Dugré et al. (2018)	Canada Cross‐sectional	50 resisters with self‐harm command hallucinations (16M/34F) 32 compliers with self‐harm command hallucinations (12M/20F) Inpatient	Mean age resisters with self‐harm command hallucinations 30.22 (5.8) Mean years since first AVH resisters with self‐harm command hallucinations 9.88 (10.45) Mean age compliers with self‐harm command hallucinations 30.38 (6.37) Mean years since first AVH compliers with self‐harm command hallucinations 10.28 (9.88)	Brief psychiatric rating scale (BPRS) DSM‐III‐R checklist Auditory Hallucinations Schedule (AHS) Family history: Childhood experiences	This study found that seriousness and frequency of childhood physical abuse, a current comorbid substance use disorder, emotional distress, general symptomatology, history of compliance and belief about compliance in the future were found to be significant risk factors of compliance with self‐harm commands in the week preceding psychiatric inpatient. Multivariate analyses revealed that severity of childhood physical abuse, belief about compliance in the future and a current comorbid substance use disorder were independent risk factors. These theory‐driven results have several important clinical implications regarding the evaluation and management of compliance with self‐harm command hallucinations. The three main risk factors showed an excellent classification accuracy, suggesting that clinicians should seek severity of childhood physical abuse, beliefs about compliance in the future and current substance use disorders. Concerning childhood traumas, while some authors stated that ‘victims are typically reluctant to disclose their histories of abuse and practitioners are often reluctant to seek it’ (Read et al. 2007), positive outcomes may result from seeking, understanding and treating the root of the client's relational problems (Ehring et al. 2014). In parallel, substance use should always be a target for prevention and treatment strategies in psychiatry. Finally, existing therapies for command hallucination hearers, focusing generally on beliefs about voices (Shawyer et al. 2012; Trower et al. 2004), could benefit from addressing compliance within an integrative framework, such as discussed earlier.
Ellett et al. (2017)	UK Cross‐sectional	151 SZ or schizophrenia affective disorder (59%M/41%F) Setting not stated	Mean age 37.23 (11.14) Mean duration of illness not stated	Beliefs About Voices Questionnaire‐Revised (BAVQ‐R)	This study found BAVQ‐R scores revealed commanding voices to be perceived as significantly more malevolent and omnipotent and to be resisted more.
Erkwoh et al. (2002)	Germany Cross‐sectional	31 with CHs; 27 paranoid SZ 3 schizoaffective disorder 1 organically caused hallucinations (21M/10F) Inpatient and outpatient	Mean age males 36.1 (not stated) Mean age females 44.4 (not stated) Mean duration of illness 63 months (not stated)	Questionnaire developed by Erkwoh et al. (2002)	This study found that characteristics comprising a voice known to the patient, emotional involvement during the hallucinations and seeing the voice as real provides significant predictivity of behaviour following command hallucinations. The comparison of compliers and non‐compliers regarding the frequency of patterns reveals that the post hallucinatory affective reaction, being alone, the assumed reality of the voice and its familiarity occur most often in compliers.
Favrod et al. (2004)	Switzerland and France Cross‐sectional	29 with AHs; 22 SZ 5 schizoaffective disorder (16M/13F) Inpatient and outpatient	Mean age 36 (11.5) Mean duration of illness not stated	Beliefs about Voices Questionnaire (BAVQ) French version	This study found that engagement and benevolence are correlated, as well as malevolence and resistance. These preliminary results, if they were to be replicated, could have some important implications for cognitive therapy. Patients who interpret their voices as being benevolent may be considered, erroneously, as requiring less therapy than patients with malevolent voices. First, the former may themselves be less willing to engage in cognitive therapy since they seek contact with their voices. This has been pinpointed by the negative correlation between the compliance factor of the Life Skills Profile and the engagement score of the Beliefs about Voices Questionnaire. Second, caretakers may assume that benevolent voices are not as bad as malevolent ones since patients do not complain about them. Consequently, they will less readily refer patients with benevolent voices to therapists. Patients with benevolent voices in therapy should be made aware of the consequences of engaging with their voices in public situations or of talking freely about their voices to other people. Therapists could involve patients in looking for alternate coping strategies to avoid negative judgement by others about their social functioning. Therapists also have to find strategies to get those patients who are attached to their voices involved in therapy.
Fenekou and Georgaca (2009)	Greece Qualitative	15 voice hearers; 8 BD 15 schizophrenia 2 schizoaffective disorder (9M/6F) Inpatient and outpatient	Age range 21–60 Mean duration of illness not stated	Semi‐structured interviews	This study found that seven of the participants, when asked how they cope with negative voices, commented that they resist them and refuse to do what the voices say. M, for example, remarked: *‘when I do not like them … when the voices are bad … when they are bad … something that wants to harm me … I do not follow them.’* The findings of this study, and of other similar studies mentioned earlier, point to the importance for clinicians to understand that voice hearers, especially those with a long‐term experience, have already elaborated explanatory frameworks for their experience as well as established coping strategies and to be prepared to work with the client in the direction of making these more functional and effective, so that the client can lead the life they wish to lead with the voices.
Fielding‐Smith et al. (2022)	UK Short‐term longitudinal study (9 days)	35 with AVHs; 12 SZ 2 schizoaffective disorder 3 other psychotic disorder 10 borderline personality disorder 3 depression with psychotic features 1 BD (11M/18F/2 other) Outpatient	Mean age 41.9 (11.4) Mean duration of illness not stated	Ecological Momentary Assessment (EMA)	This study found that momentary compliance behaviours were associated with appraisals of voice dominance and uncontrollability, with the results indicating that, on average, a unit increase in perceived voice dominance was accompanied by a 0.16‐unit increase in voice compliance. Unexpectedly, perceived voice uncontrollability was the only significant predictor of momentary resistance to voices, while voice intrusiveness was not significantly associated with either compliance or resistance behaviours. Running the reverse models indicated that levels of voice distress reported at time 1 did not significantly predict compliance or resistance at time, indicating directional effects of these behavioural responses on subsequent distress. While the results support the focus of cognitive interventions on re‐evaluating appraisals of voice power/dominance and uncontrollability, they highlight the importance of a parallel therapeutic focus on exploring and responding to negative voice content (Larøi et al. 2019).
Fox et al. (2004)	UK Cross‐sectional	32 SZ with self‐harm or harm other CHs (22M/10F) Forensic and non‐forensic mental health services	Mean age 37.2 (9.82) Mean length of time hearing hallucinations 11.72 (10.23)	Beliefs about Voices Questionnaire (BAVQ) Cognitive Assessment of Voices (CAV) Evaluative Beliefs Scale (EBS)	This study found that no significant differences were found between the compliers and non‐compliers on beliefs about benevolence or malevolence of the voice. The complier group reported that they perceived the command voice to be significantly more powerful than the non‐complier group. Further, ‘self‐harm command compliers’ reported significantly higher ratings of inferiority in social relationships, while ‘harm‐other command compliers’ reported significantly higher ratings of superiority within social relationships.
Ghadban et al. (2024)	Lebanon Cross‐sectional	61 SZ with AHs (44M/17F) Inpatient	Mean age 56.05 (12.25) Mean duration of treatment 19.69 (13.83)	Beliefs about voices questionnaire‐revised (BAVQ‐R) Arabic version Calgary depression scale for schizophrenia Arabic version Columbia suicide severity rating scale (C‐SSRS) Arabic version	This study found that higher depression was significantly associated with more omnipotence, malevolent, emotional and behavioural resistance of beliefs of voices and more psychotic symptoms, whereas lower depression was significantly associated with more benevolent, emotional and behavioural engagement with voices. Higher omnipotence and emotional resistance were significantly associated with more depression. The study found that patients with suicidal ideation had more negative and resistant beliefs about their voices, scoring higher on omnipotence, malevolent, emotional and behavioural resistance measures and lower on benevolent, emotional and behavioural engagement measures. Depression was positively associated with suicidal ideation, and beliefs about omnipotence and emotional resistance were linked to depression. Addressing depression may reduce suicidal ideation risk, while addressing maladaptive appraisals of voices may improve depression. This study emphasises the importance of addressing emotional resistance to voices in targeted interventions to reduce depression in these patients. Mental health professionals should assess not only the presence of auditory hallucinations but also the patient's beliefs about their voices, as these beliefs may significantly contribute to depression. This could guide efforts of CBT to target resistant and malevolent beliefs about voices more efficiently.
Gmeiner et al. (2020)	Austria Cross‐sectional	105 SZ or other related disorder voice hearers (59M/46F) Inpatient and outpatient	Median age 33 Median duration of voice hearing experience 10.5 years	Beliefs About Voices Questionnaire–Revised (BAVQ‐R) German version Voice Power Differential Scale (VPD) German version	This study found that there were negative correlations between the Voice Power Differential Scale (VPD) overall and the Beliefs about Voices (BAVQ‐R) subscales Benevolence (*r* = −0.268, *p* = 0.009) and Engagement‐Emotion (*r* = −0.294, *p* = 0.004) and positive correlations between VPD overall and the BAVQ‐R subscales Omnipotence (*r* = 0.485, *p* < 0.001) and Resistance‐Emotion (*r* = 0.295, *p* = 0.004).
Hacker et al. (2008)	UK Cross‐sectional	30 SZ with AVHs (22M/8F) Inpatient and outpatient	Mean age 37.6 (7.23) Mean duration of illness not stated	Beliefs about Voices Questionnaire Revised (BAVQ‐R) Cognitive Assessment of Voices Interview Schedule (CAVS) Hospital Anxiety and Depression Scale (HADS) The Safety Behaviour Questionnaire (SBQ)	This study found that safety behaviour use (e.g., compliance, appeasement and help‐seeking) (Total SB) was strongly associated with omnipotence and malevolence. However, some voice characteristics were also significantly related to safety behaviour use: degree negative content; amount negative content; and voice loudness. Anxiety was approaching significant correlation with safety behaviour use while depression was not at all significant. The significant predictors of Total SB were in order of importance: omnipotence and voice characteristics, i.e., degree of negative content and voice loudness The inclusion of safety behaviours and specific threat in the assessment of voice hearing would enhance clinical formulation and allow more targeted and effective behavioural experiments. Such experiments to test voice power are unlikely to be successful if non‐occurrence of events is attributed to subtle safety behaviours, in which the voice hearer continues to engage.
Hazell et al. (2024)	UK Cross‐sectional	208 SZ or other related disorder with AHs (150M/58F) Outpatient	Mean age 25.2 (5) Mean duration of psychosis 28.8 (17) months	Analysis of case notes	This study found two significant predictors, specifically hearing hallucinations that command the hearer to engage in self‐harm and beliefs that the voice is omnipotent. Patients were more than 20 times more likely to harm themselves if they heard self‐harm related commands than those who did not and 7 times more likely if their voice was perceived as omnipotent. A recent review indicates that some of the Evidence‐Based Risk Factors (EBRFs) (i.e., self‐harm command hallucinations) are currently neglected when assessing the risk of physical harm among psychosis patients. We suggest that the presence of these EBRFs should help in risk assessments and provide an indicator that someone may be more likely to harm themselves and/or others. However, it is important to acknowledge that these are ‘risk’ factors, not determinants. Patients reporting that their voice is making violent and/or self‐harm commands do have an increased risk of physical harm, but it is not an inevitability. Equally, it is possible that patients may physically harm themselves or others without hearing these types of command hallucinations or holding omnipotent beliefs about the voice.
Junginger (1990)	USA Cross‐sectional	44 SZ and affective disorders (gender unclear) Inpatient and outpatient	Mean age and duration of illness not stated	Psychiatric interview	This study found that the danger of complying with a hallucinatory command appeared to have less impact on actual compliance than might be expected. It was also found that recognition of the hallucinatory voice and hallucination‐related delusions appeared to have the strongest relationship with compliance.
Junginger (1995)	USA Cross‐sectional	93 SZ with a history of at least one CH (51M/42F) Inpatient	Mean age and duration of illness not stated	Premorbid adjustment scale Semi‐structured interviews	The results indicated that subjects who reported relatively less dangerous commands and subjects who could identify the hallucinated voice reported higher levels of compliance with their most recent command hallucination.
Kalhovde et al. (2014)	Norway Qualitative longitudinal (1–6 months)	14 voice hearers; 9 SZ spectrum disorders 4 other related disorder 1 Unknown diagnosis Outpatient	Median age 39 Mean duration of illness not stated	Semi‐structured interviews	The participants found ways of carrying out everyday activities by negotiating how strongly they resisted the commanding voices. One participant took a cold shower in response to the voices that demanded she throw herself in the sea on a cold winter's day. Another participant feigned cutting herself when voices persistently insisted that she hurt herself. A health care provider encouraged one participant to talk back to the voices, but the participant was unable to do so. When attempting to fall asleep while being pestered by voices, she instead contradicted the voices through imagery writing.
Lee et al. (2004)	Singapore Cross‐sectional	53 SZ with command hallucinations (24M/29F) 47 SZ without command hallucinations (26M/21F) Inpatient	Mean age SZ with command hallucinations 37.6 (9.3) Mean duration of illness SZ with command hallucinations 10 (7.7) Mean age SZ without command hallucinations 40.6 (9.9) Mean duration of illness SZ without command hallucinations 11.5 (9.2)	Semi‐structured questionnaire	This study found the significant predictors for compliance were non‐violent commands and a history of self‐harm. Patients experiencing violent command hallucinations were less likely to comply, compared with those having nonviolent command hallucinations. Of the patients who experienced command hallucinations, 19 had a history of self‐harm that was associated with compliance with command hallucinations. Clinicians should be aware that a history of self‐harm may predict compliance.
Lucas and Wade (2001)	Australia Prospective longitudinal (1 month)	30 with AHs; 24 SZ 6 schizoaffective disorders (15M/15F) Inpatient	Mean age 35.4 (13.2) Mean duration of illness 11.7 (12.9)	Beck Depression Inventory (BDI) Beliefs About the Voices Questionnaire (BAVQ) Brief Psychiatric Rating Scale (BPRS)	This study found that those who were more likely to perceive their voices as being malevolent were more likely to use resistance and less likely to use engagement. Those people who were more likely to perceive their voices as being benevolent reported less use of resistance (*r* = −0.72, *p* < 0.01) and more use of engagement (*r* = 0.84, *p* < 0.01). Malevolent voices were perceived as being more powerful (*r* = 0.45, *p* < 0.05) and benevolent voices were perceived as being less powerful (*r* = −0.30, *p* > 0.05), although this latter correlation was not significant. Higher levels of depression were associated with a greater perceived power of the voices (*r* = 0.45, *p* < 0.05). The group of people categorised as experiencing malevolent voices had significantly higher scores on the BDI than those people experiencing benevolent voices, *t*(28) = −2.4, *p* = 0.02. Those people with higher levels of psychiatric symptomatology were more likely to experience malevolent voices (*r* = 0.58, *p* < 0.01) and more likely to use resistance (*r* = 0.47, *p* < 0.01), whereas the inverse was true of benevolent voices (*r* = −0.41, *p* < 0.05) and engagement (*r* = −0.44, *p* < 0.05). If the results of the present study are shown to be robust in larger samples, this suggests that cognitive‐behaviour therapy should routinely examine ways of challenging the perceived power of the voices. This could be achieved by challenging perceived power differentials in social relationships through group identification, assertiveness training, or problem solving (Birchwood et al. 2000), or behavioural strategies that aim to test and challenge the power of the voices. This is of particular relevance to those people who experience ongoing auditory hallucinations despite compliance with antipsychotic medications, estimated to be about 40% of the population experiencing psychosis (Sheitman et al. 1998). Instead of aiming to eliminate these hallucinations, the emphasis can be on weakening critical beliefs about the power of the voices in order to alleviate distress and associated deterioration in mental functioning (Chadwick and Birchwood 1994).
Mackinnon et al. (2004)	Australia Cross‐sectional	199; 80.9% SZ 13.6% affective psychosis 3% other non‐organic psychosis 5 borderline personality disorder (134M/65F) Inpatient and outpatient	Mean age 32.7 (10.7) Mean age of onset of AHs 23.2 (10.9)	Mental Health Research Institute Unusual Perceptions Schedule (MUPS)	This study found that those unable to resist CHs rated their hallucinations as intrusive, they had fewer coping strategies (e.g., yell or talk about, talk to someone or use headphones) than those able to resist, and they were prescribed higher dosages of medication. It was also found that there was an association between the frequency with which AHs were heard and ability to resist CHs. Of the 98 who said that they could resist, 52 (53%) said they heard their voices constantly, compared with only eight (25%) of the 32 who said that they could not resist.
Marotti et al. (2025)	UK RCT	157 Latent Profile 1 (LP1) adverse voices and relational trauma (85M/70F) 84 LP2 low malevolent and omnipotent voices (58M/25F) 57 LP3 adverse voices yet low relational trauma (40M/17F) 47 LP4 high benevolent voices (29M/17F) Outpatient	Mean age LP1 adverse voices and relational trauma 41.5 (13.4) Mean duration of illness not stated Mean age LP2 low malevolent and omnipotent voices 38.7 (13.2) Mean duration of illness not stated Mean age LP3 adverse voices yet low relational trauma 37.3 (12.9) Mean duration of illness not stated Mean age LP4 high benevolent voices 37.5 (13) Mean duration of illness not stated	Beliefs About Voices Revised Questionnaire (BAVQ‐R) Trauma and Life Events (TALE) Mini‐TALE	This study found that Latent profile 1 is described as ‘Adverse voices and relational trauma’—in comparison with other profiles, individuals in this profile have the highest scores, which are higher as compared to the full sample, of fearful attachment style, trauma, and are more likely to believe that trauma and voices are related. Compared to the full sample, they report moderately higher on beliefs of voices being malevolent, omnipotent, and resisting voices, the highest scores comparative to other profiles. It has the lowest scores of benevolent voice appraisal and engagement. Latent profile 4 is described as ‘High benevolent voices’—individuals in this profile are set apart by their scoring very high as compared to the full sample and other profiles on benevolent voice appraisal and engagement with voices. Although, they are similar to the full sample, with a higher number of traumas, beliefs of voices being related to traumas and experiencing fearful attachment and higher scores of omnipotent voice appraisals. Scores of malevolence voice appraisals and resistance to voices were low, lower than the full sample scores. The current findings highlight the importance of thorough assessments of adverse experiences, alongside careful formulations of the meaning traumas have in reference to voices and how they relate to ways the voice hearer does/does not and has/has not been able to form safe attachments with others. Given profiles from a sample of individuals with distressing voices have both negative and positive voices, asking patients for detailed descriptions and interpretations of their voice could aid clinicians to not miss information, such as, benevolent appraisals and engagement with voices valued by individuals and where fewer interpersonal adversities co‐occur with distressing negative appraisals.
Morris et al. (2014)	UK Cross‐sectional	50 SZ or other related disorder with AHs (66%M/34%F) Inpatient and outpatient	Mean age 31.8 (10.54) AHs for an average of 9 years	Acceptance and Action Questionnaire (AAQ‐II) Beck Anxiety Inventory Beck Depression Inventory‐II (BDI‐II) Beliefs about Voices Questionnaire‐Revised (BAVQ‐R) Kentucky Inventory of Mindfulness Skills (KIMS) Thought Control Questionnaire (TCQ)	This study found statistically significant negative associations between psychological flexibility and nonjudgemental acceptance and appraisals of omnipotence, use of punishment thought control, level of depressive and anxiety symptoms and actions and emotions focused on resisting the voices. However, there were no relationships between psychological flexibility/nonjudgemental acceptance and distress and disruption from voices or with emotional and behavioural engagement with voices. Behavioural and emotional engagement variables both had significant positive associations with appraisals of voice benevolence and use of reappraisal thought control; emotional engagement was also negatively associated with malevolence appraisals. Finally, emotional and behavioural resistance to voices both demonstrated significant positive relationships with appraisals of voice omnipotence and malevolence, as well as use of punishment thought control. The results of this study provide evidence that psychological flexibility may be helpful for emotional problems in psychosis, while distress or disruption associated with voices specifically may be better explained by cognitive models. The findings suggest that the ability to ‘step back’ from evocative private experiences is associated with voice hearers' experiencing less depression and anxiety and engaging in fewer efforts to resist voices. It may therefore be useful to incorporate psychological flexibility and non‐judgemental awareness in clinical models of emotional distress for voice hearers as potential resilience factors.
Peters et al. (2012)	UK Cross‐sectional	46 psychosis (26M/20F) Outpatient	Mean age 36.5 (10.45) Mean duration of illness 7.4 (6.41)	Beliefs about Voices Questionnaire‐Revised (BAVQ‐R)	This study found that both omnipotence and malevolence were significantly associated with resistance, and benevolence was significantly associated with engagement. By contrast, neither omnipotence nor malevolence were associated with engagement, and benevolence was not related to resistance. Resistance was then subjected to a stepwise regression, with malevolence, omnipotence and global voice severity as independent variables. Malevolence was the only variable significantly associated with resistance. The present findings have several implications for psychological therapies. They suggest that encouraging an individual to re‐examine their voice appraisals, particularly those relating to power, may be a better way to reduce distress than trying to reduce voice activity. Similarly, working with beliefs about the intentions of the voice may be the best route to behaviour change, whether this is to reduce engagement with, for example, a commanding voice perceived as benevolent, or whether the aim is to decrease resistance to voices so as to reduce behaviours, which prevent the disconfirmation of a malevolent voice. Our findings also suggest that the goal of Cognitive Behavioural Therapy for psychosis (CBTp), and therefore the outcomes measured in CBTp trials, should not necessarily be a reduction in the severity or frequency of voices, but a change in people's appraisals and relationship with their voices (e.g., Chadwick, Sambrooke, et al. 2000), in order to reduce distress.
Rajanthiran et al. (2022)	Australia Cross‐sectional	19 SZ (11M/8F) 17 PTSD (6M/11F) 20 SZ + PTSD (8M/12F) Range of clinical settings	Mean age SZ 40.89 (13.64) Mean duration of illness SZ not stated Mean age PTSD 40.35 (13.47) Mean duration of illness PTSD notated Mean age SZ + PTSD 36.4 (13.07) Mean duration of illness SZ + PTSD not stated	Beliefs about Voices Questionnaire (BAVQ)	This study found that as a whole study population, voices were significantly more likely to be experienced as malevolent than beneficent. Participants were more likely to resist their voices than engage with them. Voice malevolence strongly correlated with emotional resistance for all three groups, *r*(17) = 0.75, *p* < 0.001. Benevolent voices strongly correlated with behavioural engagement for both the SCZ‐only and SCZ + PTSD groups, but not in the PTSD‐only group, *r*(17) = 0.17, *p* < 0.520.
Reynolds and Scragg (2010)	UK Retrospective cohort	32 with harm‐other command hallucinations; 13 SZ 1 schizoaffective disorder 14 paranoid schizophrenia 3 borderline personality disorder (32M/0F) Forensic	Mean age 34.19 (11.81) Mean age participants first heard voice 22.92 (9.82)	Mental Health Research Institute Unusual Perceptions Schedule (MUPS) Social comparison scale (SCS) Voice power differential scale (VPDS)	This study found that beliefs that the commanding voice was more powerful than the self and of a higher social rank than the self were associated with compliance. The findings of this study support cognitive models of command hallucinations (Byrne et al. 2006) and emphasise the importance of examining the relationship an individual has with the commanding voice in assessment, formulation and interventions. Within a forensic population, a high proportion of individuals who report experiencing harm‐other command hallucinations have acted on a command at some point. This highlights the need for CBT to assess harm‐other command hallucinations and challenge beliefs about the perceived power and social rank of the voices. Reviewing the beliefs an individual holds about the commanding voice and completing behavioural experiments to test out the consequences of not complying with commands might be useful. Additionally, the use of cognitive behavioural coping strategies might reduce the perceived power and social rank of the commanding voice by increasing an individual's sense of control over the commanding voice. Reducing the relative perceived power and social rank of the commanding voice could lead to a reduction in both the distress an individual experiences and the likelihood of compliance with the commanding voice.
Robles‐García et al. (2004)	Mexico Cross‐sectional	55 SZ or schizoaffective disorder with chronic AHs (38M/17F) Inpatient and outpatient	Mean age 33.42 (11.41) Mean duration of illness not stated	Beck Anxiety Inventory (BAI) Spanish version Beck Depression Inventory (BDI) Spanish version Beliefs About Voices Questionnaire (BAVQ) Spanish version	This study found that the total score in the Malevolence and Resistance subscales were positively correlated and were statistically significant with depressive symptoms evaluated with the BDI (*r* = 0.29, *p* = 0.04; *r* = 0.35, *p* = 0.01, respectively), as well as the Omnipotence subscale with the presence of anxiety symptoms according to the BAI (*r* = 0.35; *p* = 0.01).
Rogers et al. (2002)	UK Retrospective cohort	56 SZ or other related disorder non‐command hallucinators (44M/12F) 54 SZ or other related disorder command hallucinators (44M/10F) Forensic	Median age SZ or other related disorder non‐command hallucinators 33 Mean duration of illness SZ or other related disorder non‐command hallucinators not stated Median age SZ or other related command hallucinators 33 Mean duration of illness age SZ or other related command hallucinators not stated	Analysis of clinical and legal records	This study found that self‐harm command hallucinations were significant predictors of self‐harming behaviour. Should, through further replication, the content of command hallucinations predict content‐specific behaviour, then this has significant and immediate implications for current clinical practice regarding risk assessment and risk management of patients with violent or self‐harm command hallucinations. In the course of our research, we have observed that clinicians and academics alike dismiss command hallucinations as a risk indicator while citing one of the studies reviewed by Rudnick (1999). However, we urge caution with such practice until further controlled research has been conducted that examines systematically (and prospectively) the risk predictiveness of content‐specific command hallucinations in different environments.
Salim et al. (2021)	Lebanon Cross‐sectional	280 chronic SZ (180M/99F) Inpatient	Mean age 55.89 (11.27) Mean duration of illness 28.87 (12.34)	Beliefs about Voices Questionnaire‐Revised (BAVQ‐R) Arabic version Positive and Negative Syndrome Scale (PANSS) Arabic version Voice Compliance Scale (VCS) Arabic version	This study found that the PANSS subscales scores were significantly associated with higher compliance to voices. A higher resistance to beliefs about voices was significantly associated with lower compliance to voices. These findings should motivate clinicians in Lebanon to apply specialised therapies for commanding voices (CV), such as cognitive therapy, which was proven to significantly reduce CV (Trower et al. 2004). Teaching patients behavioural management techniques can successfully decrease the percentage of compliance to CV towards self or others (Buccheri et al. 2007). Furthermore, during recent years, modifying cognitive behavioural therapy to target specific subtypes of auditory hallucinations such as CV was found to be an efficient intervention (Smailes et al. 2015).
Sayer et al. (2000)	UK Longitudinal (4 weeks)	26 SZ with AVHs (13M/13F) Inpatient and outpatient	Mean age 37.6 (not stated) Mean duration of illness 15.1 (not stated)	Beliefs About Voices Questionnaire (BAVQ)	This study found positive relationships between a resistive coping style and an attribution of malevolence to voices, and between an engaging coping style and an attribution of benevolence to voices. Coping and attributional styles were not necessarily stable over time. The use of a panel design allowed for measurement of temporal variations in the respondents' beliefs and coping styles. The investigator found that both may change over time. This indicates that irrespective of the treatment regimen, any form of psychological treatment should be based on regular assessment and must be flexible according to the fluctuating needs of the individual in treatment.
Shawyer et al. (2007)	Australia Cross‐sectional	41 with command hallucinations; 73% SZ 20% schizoaffective disorder 7% mood disorder with psychotic features (22M/19F) Private and public mental health services	Mean age 40 (10) Mean duration of hearing voices 15.8 (11)	Voices Acceptance and Action Scale (VAAS)	This study found that those who reported not having complied with harmful commands in the previous 6 months had a higher action score (autonomous action is defined as behaviour that is self‐directed rather than being a reaction to the voices) than those who did comply.
Shawyer et al. (2008)	Australia Retrospective	50 community participants with SZ or other related disorder (33M/17F) 25 forensic participants with SZ or other related disorder (23M/2F) Forensic, inpatient and outpatient	Mean age community participants with SZ or other related disorder 37.1 (10.4) Mean age forensic participants with SZ or other 33.6 (10.4) Mean duration of auditory hallucinations before index CH for both groups 8.4 (9.4)	Beliefs about the Voices Questionnaire Revised (BAVQ‐R) State–Trait Anger Expression Inventory‐2 (STAXI)	This study found that compliance was associated with increasing age, viewing the command hallucination as positive, congruent delusions, and reporting low maternal control in childhood. Antipsychotic medication was protective while, contrary to expectations, traditional predictors of violence reduced the odds of compliance with command hallucinations viewed as threatening. Omnipotence (OR = 1.28, CI = 1.04–1.57, *p* = 0.02) and trait anger (OR = 1.17, CI = 1.00–1.35, *p* = 0.04) significantly predicted compliance. Those who were not taking antipsychotic medication at the time of the Index CH were more likely to comply. All participants who described the experience of the Index command as positive complied either partially or fully. Absence of other harmful command hallucinations around the time of the Index CH also increased likelihood of compliance: 12 (75%) of the noncompliers heard other dangerous command hallucinations compared with 9 (39%) partial compliers and 13 (36%) full compliers. Only 3 of the 18 (17%) total compliers had a history of violence compared with 7 (40%) noncompliers and 6 (54%) partial compliers.
Simms et al. (2007)	UK Cross‐sectional	17 patients with SZ and a history of self‐harm (53%M/47%F) 16 patients with SZ and without a history of self‐harm (81%M/19%F) Inpatient	Mean age patients with SZ and a history of self‐harm 37.5 (8.4) Mean duration of illness patients with SZ and a history of self‐harm 13.8 (8) Mean age patients with SZ and without a history of self‐harm 32.8 (14.7) Mean duration of illness patients with SZ and without a history of self‐harm 9 (14.5)	Beck Depression Inventory (BDI) Beck Suicide Intent Scale (BSI) Beliefs About Voices Questionnaire Revised (BAVQ‐R)	This study found that those who experience verbal auditory hallucinations and engage in acts of self‐harm experience significantly more malevolent beliefs about their voice and tend to resist their voice more strongly than those who have no history of self‐harm (*p* = 0.012 and *p* = 0.024). There was a significant negative association between symptoms of depression and benevolence (*r* = −0.593; *p* < 0.05), and depression and engagement (*r* = −0.602; *p* < 0.05).
So and Wong (2008)	Hong Kong Cross‐sectional	22 FEP with persistent AHs (6M/16F) Outpatient	Mean age 20.6 (4.3) Mean duration of experiencing AHs 28 (21) months	Beliefs About Voices Questionnaire–Revised (BAVQ‐R) Semi‐structured Interview Schedule	This study found that malevolence was correlated with omnipotence (*r* = 0.46, *p* = 0.03), emotional resistance (*r* = 0.64, *p* = 0.001), and frequency of AHs (*r* = −0.51, *p* = 0.02). Benevolence was correlated with emotional engagement (*r* = 0.81, *p* < 0.001) and positivity of content of AHs (*r* = 0.46, *p* = 0.04). Omnipotence was correlated with emotional resistance (*r* = 0.58, *p* = 0.01) and volume of AHs (*r* = 0.42, *p* = 0.05).
So et al. (2016)	The Netherlands Cross‐sectional	40 SZ or other related disorder with persistent AVHs (8M/32F) 135 non‐clinical voice hearers (43M/92F) 126 HCs (40M/86F) Outpatient	Mean age SZ or other related disorder with persistent AVHs 45.43 (11.95) Mean age non‐clinical voice hearers 50.6 (12.7) Mean age HCs 50.8 (14.52) Mean duration of illness unclear	Beliefs about Voices Questionnaire‐Revised (BAVQ‐R) Child Trauma Questionnaire–Short Form (CTQ‐SF) Psychotic Symptom Rating Scales (PSYRATS) The revised NEO Personality Inventory (NEO–PI–R)	This study found that in both clinical and non‐clinical voice‐hearers alike, a higher level of neuroticism was associated with more distress and behavioural resistance in response to auditory verbal hallucinations, as well as a stronger tendency to perceive voices as malevolent and powerful. Malevolence and power significantly mediated the association between levels of neuroticism and distress and the association between levels of neuroticism and resistance.
So et al. (2021)	Hong Kong Short‐term longitudinal study (6 days)	41 SZ spectrum disorders with frequent AVHs (15M/26F) Outpatient	Mean age 43.83 (12.4) Mean duration of illness unclear	Ecological Momentary Assessment (EMA) Beliefs about Voices Questionnaire–Revised (BAVQ‐R) Chinese version	This study found that Malevolence and Omnipotence were positively associated with each other, *r*s(39) = 0.50, *p* = 0.001, and with Resistance, *r*s(39) = 0.53, *p* < 0.001, and *r*s(39) = 0.38, *p* = 0.015, respectively, whereas Benevolence was positively associated with Engagement, *r*s(39) = 0.82, *p* < 0.001, and negatively associated with Resistance, *r*s(39) = −0.41, *p* = 0.008. Omnipotence was also positively associated with Engagement, *r*s(39) = 0.33, *p* = 0.037.
Soppitt and Birchwood (1997)	UK Cross‐sectional	21 chronic SZ (16M/5F) Outpatient	Mean age 43 (not stated) Mean duration of hallucinosis 18	Beck Depression Inventory (BDI) Beliefs About Voices Questionnaire (BAVQ) Classification of Derogatory and Non‐derogatory Content	This study found that depression was linked to both derogatory voice content (*p* = 0.0004) and malevolent beliefs and/or resistance coping strategies (*p* = 0.0176) as well as voice intrusiveness and loudness. A comparison of these sample means showed significantly higher levels of depression in those with malevolent hallucinations. This was in keeping with previous work showing that beliefs are linked with depression (Chadwick and Birchwood 1994, 1995). There are clinical implications from this work. Drake and Cotton (1986) have shown that the suicide rate of 10% in the schizophrenic population occurs almost exclusively in association with depression. Clinicians should therefore screen for depression and suicidal risk in those schizophrenics who present with derogatory verbal hallucinoses.
Stephanie et al. (2018)	Australia Cross‐sectional	62 voice hearers; 48.4% SZ 17.7% schizoaffective disorder 19.4% BD 14.5% major depressive disorder (40.3%M/59.7%F) Inpatient and outpatient	Mean age 40.23 (12.94) Mean duration of illness 15.05 (11.12)	Beliefs About Voices Questionnaire–Revised (BAVQ‐R) Psychotic Symptoms Rating Scales–Auditory Hallucinations (PSYRATS) Southampton Mindfulness of Voices Questionnaire (SMVQ)	This study found significant negative associations between mindfulness of voices (SMVQ) and voice related distress (PSYRATS) and resistance to voices (BAVQ‐R). Additionally, when scores on items relating to emotional resistance were removed and only behavioural resistance was examined, this significant negative relationship remained. Analyses revealed significant positive associations between loudness of voices and voice‐related distress, frequency and duration of voices and voice‐related disruption and anxiety and resistance to voices. However, when emotional resistance items were removed, the relationships with behavioural resistance were no longer significant. Clinically, these findings indicate that provision of interventions aimed at assisting clients to become more mindful of experienced voices may be helpful in relation to managing the negative emotional experience of voices, but that addressing mindfulness alone may not be sufficient to produce changes in client functioning (Morris et al. 2014). Consequently, mindfulness should perhaps not be seen as a stand‐alone intervention for persistent voices but may be more beneficial when delivered in conjunction with broader rehabilitation and recovery interventions.
Suryani et al. (2013)	Indonesia Qualitative	13 SZ who have experienced AHs (6M/7F) Outpatient	Age range 19–56 Duration of AHs estimated to be over 4 years	In‐depth focus interviews	This study found that for a number of the participants, life at times was robotic in nature, as they felt devoid of control and powerless to resist the voice commands irrespective of whether they were positive or negative in nature as described by one of the participants: *the voices seemed to command my brain … … In my mind I felt as if I was under their command*. One of the participants gave an example of how the voices exerted power over him. He said: *I remember that after recovering and going home 2 years ago, the voices instructed me to climb a mountain. At the time, I just followed their instruction. I could not reject it …*.
Trower et al. (2004)	UK Longitudinal RCT (12 months)	18 SZ or other related disorder receiving cognitive therapy for CHs (10M/8F) 20 SZ or other related disorder receiving TAU (14M/6F) Forensic	Mean age SZ or other related disorder receiving cognitive therapy for CHs 36.6 (10.3) Mean duration of voices SZ or other related disorder receiving cognitive therapy for CHs 13.4 (9.9) Mean age SZ or other related disorder receiving TAU 35.1 (10.4) Mean duration of voices SZ or other related disorder receiving TAU 10 (5.7)	Calgary Depression Scale for Schizophrenia (CDSS) Cognitive Assessment Schedule (CAS) Omniscience Scale Psychotic Symptom Rating Scales (PSYRATS) Voice Compliance Scale (VCS) Voice Power Differential scale (VPD)	This study found that the Cognitive Therapy for Command Hallucinations (CTCH) and TAU groups did not differ in compliance with commands at baseline, as measured by the Voice Compliance Scale. Large and significant reductions in compliance behaviour were obtained favouring the cognitive therapy group. Improvements were also observed in the CTCH but not the control group in degree of conviction in the power and superiority of the voices and the need to comply, and in levels of distress and depression. No change in voice topography (frequency, loudness, content) was observed. The differences were maintained at 12 months' follow‐up. Perhaps more importantly, the risk factors for compliance in the CTCH group had reduced markedly, particularly the perceived power of the voice, its omniscience and controllability, and the need to appease it (14% of the CTCH group were appeasing or complying v. 53% of the TAU group). CTCH has a comparatively large effect in reducing compliance with commands and delusional distress. CTCH significantly reduces the impact of ‘power’ beliefs which, according to our theory, have a causal role in compliance and are therefore a risk factor. CTCH is the first practical intervention that we know of that has a specific effect on compliance with command hallucinations.
van der Gaag et al. (2003)	The Netherlands Cross‐sectional	54 voice hearers; 78% SZ 13% affective disorders 9% personality disorders (56%M/44%F) Inpatient and outpatient	Mean age 39.6 (12.3) Median duration of illness 11	Beck Depression Inventory (BDI) Beliefs about Voices Questionnaire (BAVQ) Spielberger State–Trait Anxiety Scale (SSTAS) Dutch state version	This study found that voices perceived as malevolent were resisted (*r* = 0.71) and not engaged (*r* = −0.67), whereas benevolent voices were engaged (*r* = 0.83) and not resisted (*r* = −0.64). Malevolent beliefs were linked to higher distress. Those holding benevolent beliefs experienced less distress. From this study, it can be concluded that a change in beliefs about voices can make a difference for patients. Although voice activity might continue, patients' distress could be eased, theoretically at least. Changes in appraisal can be accomplished with cognitive behaviour therapy.
Zanello and Dugré (2021)	Switzerland Cross‐sectional	5 SZ or other related disorder with high benevolent and low malevolent beliefs about voices (2M/3F) 39 SZ or other related disorder with low benevolent and high malevolent beliefs about voices (24M/15F) 8 SZ or other related disorder with high benevolent and malevolent beliefs about voices (5M/1F) 26 SZ or other related with low benevolent and malevolent beliefs about voices (18M/8F) Outpatient	Mean age SZ or other related disorder with high benevolent and low malevolent beliefs about voices 43 (9.6) Mean duration of illness SZ or other related disorder with high benevolent and low malevolent beliefs about voices 16.8 (10.3) Mean age SZ or other related disorder with low benevolent and high malevolent beliefs about voices 38.1 (10.7) Mean duration of illness SZ or other related disorder with low benevolent and high malevolent beliefs about voices 11.5 (7.6) Mean age SZ or other related disorder with high benevolent and malevolent beliefs about voices 38.5 (10.7) Mean duration of illness SZ or other related disorder with high benevolent and malevolent beliefs about voices 10.3 (9.9) Mean age SZ or other related with low benevolent and malevolent beliefs about voices 39.77 (10.2) Mean duration of illness SZ or other related with low benevolent and malevolent beliefs about voices 13.4 (8.8)	Beliefs About Voices Questionnaire–Revised (BAVQ‐R) French version	This study found that high benevolence and low malevolence scores has greater engagement score than low benevolence and high malevolence. Furthermore, high benevolence and low malevolence had significantly higher omnipotence and resistance scores than low benevolence and high malevolence and high benevolence and malevolence. As found in this study, it seems that some patients perceive their AVHs as both malevolent and benevolent (approximately 10% of the total sample size). Despite that we hypothesise that these AVHs would be difficult to treat due to the patient's ambivalence regarding treatment; studies on treatments for AVHs should investigate whether the heterogeneity of voice intents may interfere with treatments or may be specific to a subgroup. Second, as observed in the current study, earlier findings have suggested that beliefs about AVHs are stable across time (from 6 weeks to a year, irrespectively of treatments) (Csipke and Kinderman 2006; Hartigan et al. 2014). Given this rigidity, it concurs with the fact that the psychological treatments should focus on patient's phenomenology (e.g., belief system) rather than on symptoms (e.g., distress). It has been proposed that this should reduce the severity of AVHs but also modify the relation between the patients and their voices (Hayward et al. 2017). In addition, identifying sequential changes between malevolence/benevolence (in patients reporting both high malevolence and benevolence) and their affective and behavioural responses may increase our understanding of relationships between patients and their voices.

Abbreviations: AH, auditory hallucinations; AVHs, auditory verbal hallucinations; BD, bipolar disorder; CBT, cognitive behaviour therapy; CI, confidence interval; DSM, Diagnostic and Statistical Manual of Mental Disorders; F, female; FEP, first episode psychosis; HCs, healthy controls; M, male; OR, odds ratio; RCT, randomised controlled trial; SD, standard deviation; SZ, schizophrenia; TAU, treatment as usual.

### 3.1 Risk of Bias and Certainty Assessment

In accordance with the EPHPP tool, the global quality rating for the included studies was rated as ‘strong’ (*n* = 34), ‘moderate’ (*n* = 14) and ‘weak’ (*n* = 3) (See Table 2). For the five included qualitative studies, the global quality rating according to the JBI tool was rated as ‘strong’ (*n* = 3) and ‘moderate’ (*n* = 2) (see Table 3). This indicates that most of the included studies were characterised by low risk of bias.

## 4 Compliance

### 4.1 Benevolence

Benevolence refers to the perception that a hallucinated voice is kind, protective or has helpful intentions towards the individual. Seventeen studies and qualitative evidence consistently showed that voices appraised as benevolent were more likely to be complied with (Chadwick and Birchwood 1994; Shawyer et al. 2008; see Table 4). This effect likely reflects the tendency for individuals to trust benevolent voices and regard their commands as supportive or in their best interests. These results highlight the clinical importance of addressing benevolence beliefs, as voices appraised as protective or helpful may be particularly difficult to resist, potentially elevating the risk of harmful behaviour.

### 4.2 Omnipotence

Omnipotence refers to the belief that a voice possesses unlimited power or the ability to exert control over events, outcomes or the voice‐hearer's life. Nine out of eleven studies investigating the association between omnipotence and compliance found significant positive correlations, indicating that voices appraised as powerful were more likely to be complied with (Birchwood et al. 2018; Bucci et al. 2013; Fox et al. 2004; Hacker et al. 2008; Hazell et al. 2024; Reynolds and Scragg 2010; Shawyer et al. 2008; So et al. 2021; Trower et al. 2004). Specifically, individuals were seven times more likely to engage in self‐harm if voices were deemed omnipotent (Hazell et al. 2024). Complementing this, a qualitative study reported that participants who felt powerless did not believe that they could disobey their voices, which often led to compliance behaviours (Suryani et al. 2013). However, one study found no association between omnipotence and compliance, which may reflect a failure to distinguish between voices genuinely appraised as powerful and voices that merely claimed power without the hearer endorsing this belief (Peters et al. 2012).

### 4.3 Omniscience

Omniscience refers to the perception that a voice is all‐knowing. One study found a significant positive correlation between omniscience and compliance (Birchwood and Trower 2006). Additionally, voices rated as powerful were more likely to be rated as omniscient (Birchwood et al. 2004), suggesting some overlap between perceptions of power and knowledge. Taken together, these findings indicate that voices perceived as powerful (omnipotent) or all‐knowing (omniscient) are more likely to elicit compliance behaviours.

### 4.4 Negative Consequences and Future Compliance

One study found that the perceived negative consequences of disobeying voice commands were significantly associated with compliance (Barrowcliff and Haddock 2010). Similarly, a qualitative study reported that participants' compliance was influenced by beliefs about the effects of transgression (Beck‐Sander et al. 1997). In addition, individuals who believed that they would comply with commands in the future were more likely to comply with current voice commands (Dugré et al. 2018). Together, these findings suggest that compliance may be influenced both by fear of negative outcomes and by expectations regarding the likelihood of future compliance.

### 4.5 Attentive Awareness

Mindfulness of voices, defined as attentive awareness of the voice (Chadwick et al. 2007), has been examined in relation to compliance with CHs. Greater attentive awareness was associated with increased compliance behaviours (Chadwick et al. 2007; Stephanie et al. 2018). These findings were observed in samples with a mean illness duration of over 14 years, suggesting that the association may not generalise to individuals in the early stages of psychosis.

### 4.6 Trauma

Three studies investigated the association between childhood trauma and compliance with CHs (Birchwood et al. 2018; Dugré and West 2019; Dugré et al. 2018). Childhood physical abuse, emotional abuse and emotional neglect were each found to significantly predict compliance to CHs (Birchwood et al. 2018; Dugré and West 2019; Dugré et al. 2018). These findings suggest that experiences of past physical and emotional abuse, as well as emotional neglect, may increase the likelihood of engagement with commands. However, one study found that the effect of these trauma subtypes on compliance was mediated by omnipotence beliefs, indicating that trauma may influence compliance indirectly through cognitive appraisals rather than directly (Birchwood et al. 2018). These findings highlight the need for further research to clarify how different trauma experiences contribute to compliance, both directly and via cognitive mediators such as beliefs about voice power.

### 4.7 Social Rank

Social rank refers to the perceived relative position or status of the voice‐hearer to the voice, as well as to others in their social relationships. Four studies examined the association between social rank and compliance (Barrowcliff and Haddock 2010; Fox et al. 2004; Reynolds and Scragg 2010; Trower et al. 2004). Voices perceived to be of a higher social rank in comparison to the voicer‐hearer significantly predicted compliance with commands (Barrowcliff and Haddock 2010; Reynolds and Scragg 2010). Conversely, reductions in the perceived superiority of the voice significantly led to a reduction in compliance (Trower et al. 2004). Additionally, compliance with self‐harm CHs was associated with higher levels of inferiority within social relationships (Fox et al. 2004). These findings suggest that individuals who perceive themselves as inferior in comparison to their voices and/or interpersonal relationships were more likely to comply with voice commands. In contrast, compliance with harm‐other CHs was significantly associated with higher levels of perceived superiority within social relationships (Fox et al. 2004). These findings indicate that perceptions of relative social rank, both in relation to the voice and social environment, play a key role in determining compliance.

### 4.8 Voice Identity

Voice identity refers to the perception of the voice as a distinct entity, often with a recognisable personality, gender, age or familiarity to the voice‐hearer. Qualitative findings regarding voice identity and compliance have been mixed. While voices appraised as God were consistently associated with greater compliance (Chadwick and Birchwood 1994; Beck‐Sander et al. 1997), findings regarding voices appraised as the Devil were mixed, with some studies linking them to resistant behaviours (Chadwick and Birchwood 1994) and others to increased compliance and feelings of loss of control (Beck‐Sander et al. 1997). These differences may reflect variations in the subjective meaning or perceived threat of the voices, highlighting the need for further research to clarify how voice identity influences compliance (Chadwick and Birchwood 1994; Beck‐Sander et al. 1997).

### 4.9 Voice Familiarity

Voice familiarity refers to the extent to which a voice is perceived as known or recognisable to the voice‐hearer, such as resembling a real person from their past or present. Four studies examined the association between voice familiarity or recognition and compliance with commands (Chadwick and Birchwood 1994; Erkwoh et al. 2002; Junginger 1990; Junginger 1995). These studies investigated whether individuals were more likely to comply when the commanding voice was perceived as familiar (i.e., resembling a parent, friend or authority figure) compared to when it was unfamiliar or anonymous. Three studies reported a significant positive correlation between voice familiarity and compliance (Erkwoh et al. 2002; Junginger 1990; Junginger 1995), suggesting that recognition or perceived familiarity may increase the likelihood of compliance. These findings underscore the need for further research to clarify the mechanisms through which familiar voices influence compliance.

### 4.10 Voice Content

Four studies investigated the voice content of CHs (Chadwick and Birchwood 1994; Hacker et al. 2008; Hazell et al. 2024; Junginger 1995). Qualitative studies found that participants were more likely to comply with mild rather than severe commands (Chadwick and Birchwood 1994), and similarly, less dangerous (Junginger 1995) and non‐violent CHs (Lee et al. 2004) predicted higher compliance. Additionally, individuals experiencing self‐harm commands were found to be 20 times more likely to harm themselves compared to those who did not hear such commands (Hazell et al. 2024). Overall, these findings indicate that compliance is influenced by the content of the voice, with both the severity and the specific nature of the command influencing behavioural responses.

### 4.11 Anger, Impulsivity and Social Isolation

Two studies reported a significant positive correlation between anger and compliance, suggesting that heightened emotional arousal may increase the likelihood of obeying voice commands (Bucci et al. 2013; Shawyer et al. 2008). Impulsivity was also found to significantly predict compliance in one study, indicating that individuals may respond to commands without fully considering the potential consequences (Bucci et al. 2013). Additionally, being alone at the time of voice hearing was associated with higher compliance in one study (Erkwoh et al. 2002). Overall, these findings suggest that emotional and situational factors, including anger, impulsivity and social isolation, may contribute to compliance with CHs.

### 4.12 Feelings of Having to Obey

Two studies investigated the relationship between feelings of obligation to obey CHs and compliance (Dugré and West 2019; Dugré et al. 2018). Both studies reported a significant positive association, indicating that stronger feelings of having to obey the voice were associated with higher levels of compliance (Dugré and West 2019; Dugré et al. 2018). These findings suggest that the perceived obligation to comply is an important predictor of behavioural responses to CHs.

### 4.13 Substance Use Disorder (SUD)

One study found that individuals with a comorbid SUD were more likely to comply with CHs (Dugré et al. 2018). These findings indicate a positive association between SUD and compliance behaviour, possibly by increasing disinhibition and impulsivity, which may in turn increase the likelihood of acting on CHs.

### 4.14 Hallucination‐Related Delusions

Two studies investigated the relationship between hallucination‐related delusions and compliance with CHs (Junginger 1990; Shawyer et al. 2008). Both reported a significant positive correlation, indicating that individuals who experienced delusional beliefs related to their hallucinations were more likely to comply with commands. This finding is further supported by a qualitative study showing that individuals with delusions tended to comply with commands consistent with their delusional content (Beck‐Sander et al. 1997). These findings suggest that the presence of hallucination‐related delusions increases the likelihood of compliance, particularly when commands are consistent with the content of the delusional beliefs.

### 4.15 Medication Non‐Adherence

One study found that individuals who were not taking antipsychotic medication were significantly more likely to comply with CHs (OR = 5.37, *p* = 0.002) compared with those taking medication (Shawyer et al. 2008). These findings suggest that antipsychotic treatment may reduce the likelihood of compliance and that medication non‐adherence could increase engagement with CHs.

### 4.16 Symptom Severity and Hospitalisation

Compared to patients who were non‐compliant with their voices, patients who were compliant were characterised by significantly higher positive and negative symptoms and greater length of hospitalisation, which may be suggestive of greater overall illness severity (Salim et al. 2021). This is further supported by evidence that compliant patients received significantly higher mean doses of antipsychotic medication than those who resisted their CHs, providing additional indication of greater illness severity (Mackinnon et al. 2004).

## 5 Appeasement

### 5.1 Perceived Dangerousness

Appeasement refers to the partial or negotiated compliance intended to minimise the perceived threat posed by the voice. Individuals were significantly more likely to appease commands deemed to be dangerous (Junginger 1995). As appeasement has been less extensively investigated than full compliance, further research is needed to better understand the factors influencing this response.

### 5.2 Self‐Harm

A qualitative study found that individuals with CHs reported using self‐harm to cope with their voices, both as a form of emotional regulation and as a way to appease voices, aiming to reduce the perceived threat or make them stop (Denno et al. 2022). These findings indicate that appeasement can involve self‐directed harm, highlighting the high‐risk potential of some appeasement strategies.

### 5.3 Harm‐Other Commands

A qualitative study found that individuals tended to appease their harm‐other commands by engaging in acts of self‐harm, such as cutting one's wrist when the voice instructed them to attack a member of staff or ingesting harmful substances in order to appease a voice's sexualised commands (Beck‐Sander et al. 1997). Participants were found to be more willing to comply with commands to harm themselves than to harm others (Beck‐Sander et al. 1997). Complementing this, higher rates of partial compliance were observed for commands directing harm at others compared to self‐harm, whereas no partial compliance occurred for benign commands (Barrowcliff and Haddock 2010). Together, these findings indicate that partial or self‐directed acts may function as appeasement strategies in response to harm‐other commands. However, further research is needed to clarify the relationship between harm‐other commands, appeasement and self‐harm.

### 5.4 Mild Versus Severe Commands

Appeasement refers to the strategy of complying with less severe or mild voice commands in order to avoid or mitigate more severe demands. One study reported that 10 of 12 voices that issued severe commands also gave mild commands, and in all 10 cases, these milder commands were obeyed at least occasionally (Chadwick and Birchwood 1994). These findings indicate that compliance with mild commands often occurs alongside compliance with more severe commands, suggesting a potential pattern of appeasement.

### 5.5 Avoiding Provocation

A recent study found that other coping strategies included deliberately complying with or appeasing auditory verbal hallucinations (AVHs) (8 of 25 and 6 of 10 participants, respectively), avoiding provoking voices and avoiding situations in which losing control could be dangerous (Denno et al. 2022). Most participants reported being able to negotiate with or overcome voices some of the time, depending on situational factors, mood and the intensity of the voices (Denno et al. 2022). Together, these findings indicate that appeasement is a commonly used strategy, often occurring alongside other coping approaches, and may vary according to context and voice characteristics.

## 6 Resistance

### 6.1 Malevolence

Twenty‐four studies investigated the association between malevolence and resistance to CHs, and all reported a significant positive correlation (see Table 4). Appraisals of malevolence were significantly associated with both emotional and behavioural resistance to voices (Chawla et al. 2019). In addition, four studies found that malevolence was significantly positively correlated with depression (Ghadban et al. 2024; Lucas and Wade 2001; Robles‐García et al. 2004; Soppitt and Birchwood 1997). Overall, these findings indicate that perceiving voices as malevolent consistently predicts both emotional and behavioural resistance and is also associated with higher levels of depression.

### 6.2 Omnipotence

Thirteen studies examined the association between omnipotence and resistance, with all reporting significant positive correlations (Andrew et al. 2008; Birchwood et al. 2004; Chawla et al. 2019; Ellett et al. 2017; Ghadban et al. 2024; Gmeiner et al. 2020; Marotti et al. 2025; Morris et al. 2014; Peters et al. 2012; So and Wong 2008; So et al. 2016; So et al. 2021; Zanello and Dugré 2021). In contrast, nine of 11 studies found a positive correlation between omnipotence and compliance (Birchwood et al. 2018; Bucci et al. 2013; Fox et al. 2004; Hacker et al. 2008; Hazell et al. 2024; Reynolds and Scragg 2010; Shawyer et al. 2008; So et al. 2021; Trower et al. 2004). These apparently conflicting findings may reflect sample differences: Individuals in forensic samples tended to obey powerful voices, whereas those in non‐forensic samples were more likely to resist them. This may suggest that people who are unable to resist omnipotent voices are more likely to enter forensic services, while those who are able to resist are less likely to come into contact with such settings.

### 6.3 Perceived Control

Two studies investigated the association between perceived control over CHs and resistance (Beck‐Sander et al. 1997; Birchwood and Trower 2006). Both studies found that greater perceived control over commands was associated with increased likelihood of resisting CHs (Beck‐Sander et al. 1997; Birchwood and Trower 2006). These findings suggest that individuals who believe they can exert control over their voices are more likely to resist commands.

### 6.4 Suicidal Ideation

One study found that suicidal ideation was significantly positively associated with resistance to CHs (Ghadban et al. 2024). This suggests that individuals experiencing suicidal thoughts may be more likely to resist acting on commands. However, given the limited evidence, further research is needed to clarify the mechanisms underlying this relationship.

### 6.5 Trauma

Two studies reported that experiencing multiple childhood traumas, interpersonal adversities and having a fearful attachment style were positively associated with resistance to CHs (Begemann et al. 2022; Marotti et al. 2025). In addition, traumatic experiences were associated with malevolent and omnipotent appraisals of voices (Andrew et al. 2008; Begemann et al. 2022; Marotti et al. 2025), suggesting that trauma may shape how individuals interpret and respond to CHs.

### 6.6 Intrusiveness and Authoritativeness

Perceptions of higher intrusiveness and lower authoritativeness were associated with greater resistance to CHs (Mackinnon et al. 2004). These findings suggest that voices perceived as less authoritative and more intrusive may motivate individuals to resist commands. However, given the limited research on this topic, further studies are needed to clarify the role of intrusiveness and authoritativeness in compliance, appeasement and resistance.

### 6.7 Frequency and Loudness

Three studies have investigated voice topography in relation to resistance to CHs, focusing on frequency and loudness (Andrew et al. 2008; Mackinnon et al. 2004; Soppitt and Birchwood 1997). Both greater voice frequency and louder voices were associated with increased resistance, with louder voices also positively correlated with malevolent appraisals (Soppitt and Birchwood 1997). These findings suggest that more prominent and intense voices may elicit stronger resistance to commands.

## 7 Discussion

This systematic review synthesised the clinical correlates of compliance, appeasement and resistance to CHs, highlighting overlapping and distinct determinants and their implications for clinical practice. Across all response types, cognitive appraisals of voices—particularly beliefs about omnipotence, malevolence or benevolence—emerged as central determinants of behaviour, consistent with the *Cognitive Model of Voices* (Chadwick and Birchwood 1994; Beck‐Sander et al. 1997). Emotional, relational, behavioural, trauma‐related and symptom severity factors further modulated these responses, providing targets for assessment and intervention.

**FIGURE 2:**
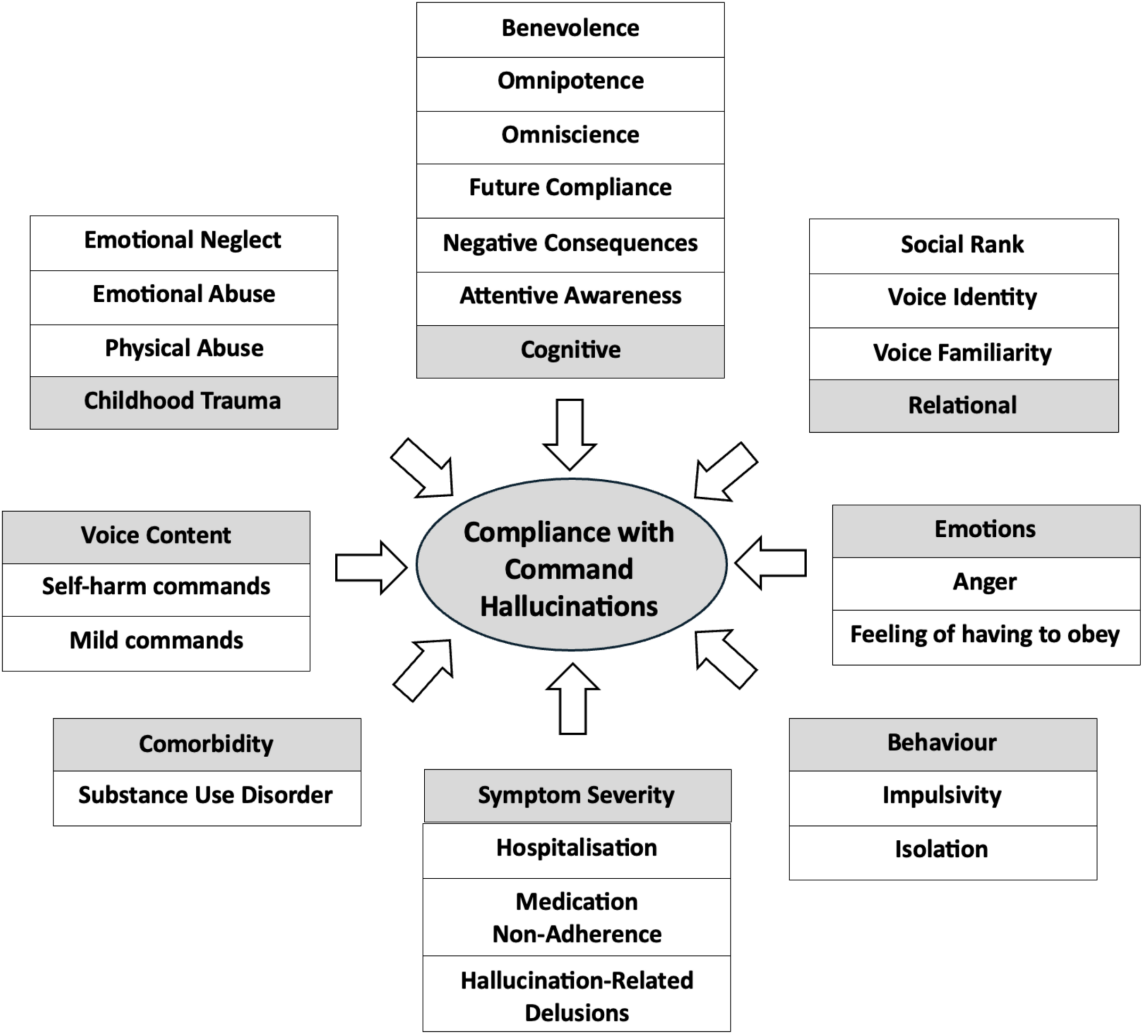
Factors associated with compliance with command hallucinations.

**FIGURE 3:**
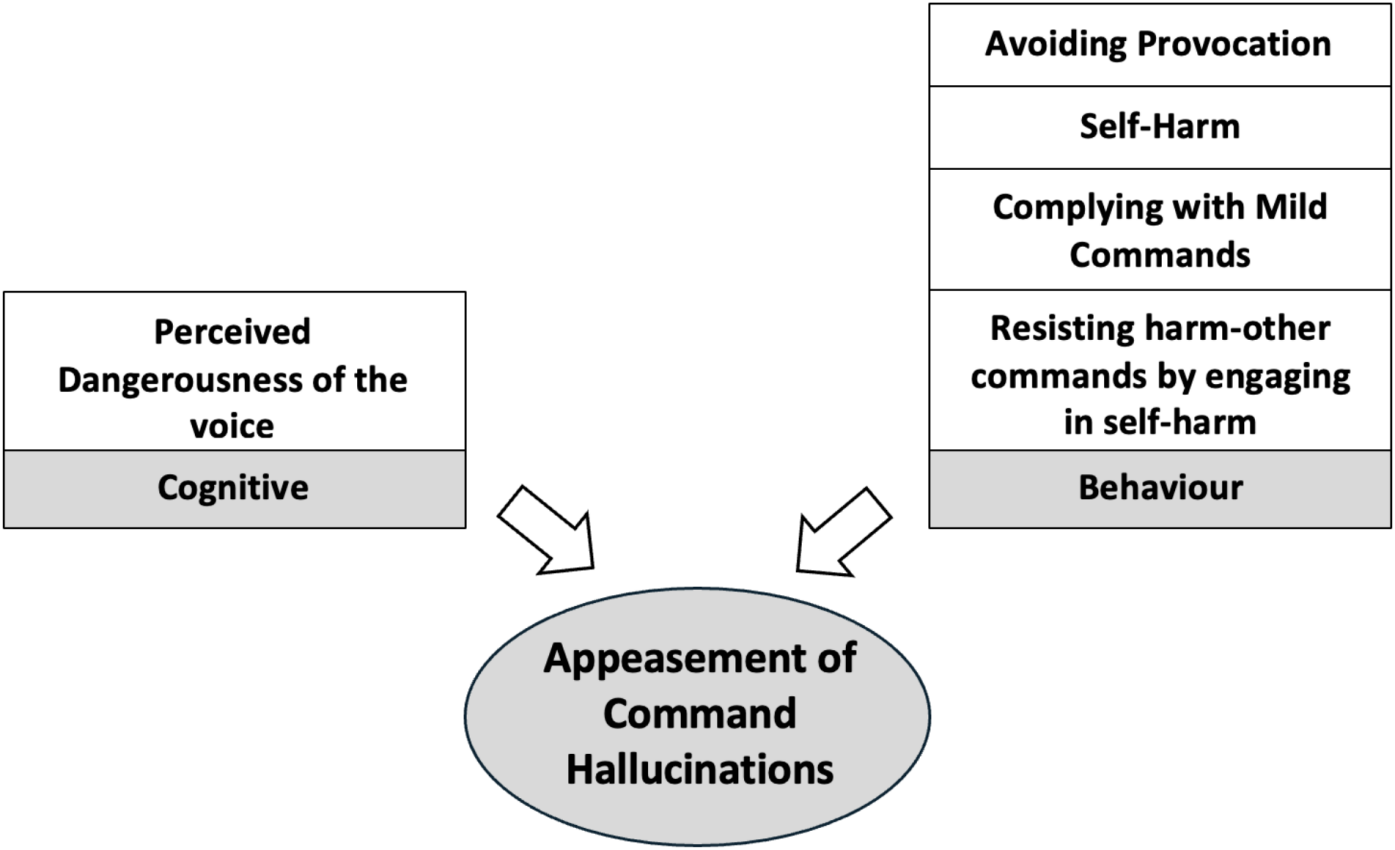
Factors associated with appeasement with command hallucinations.

**FIGURE 4:**
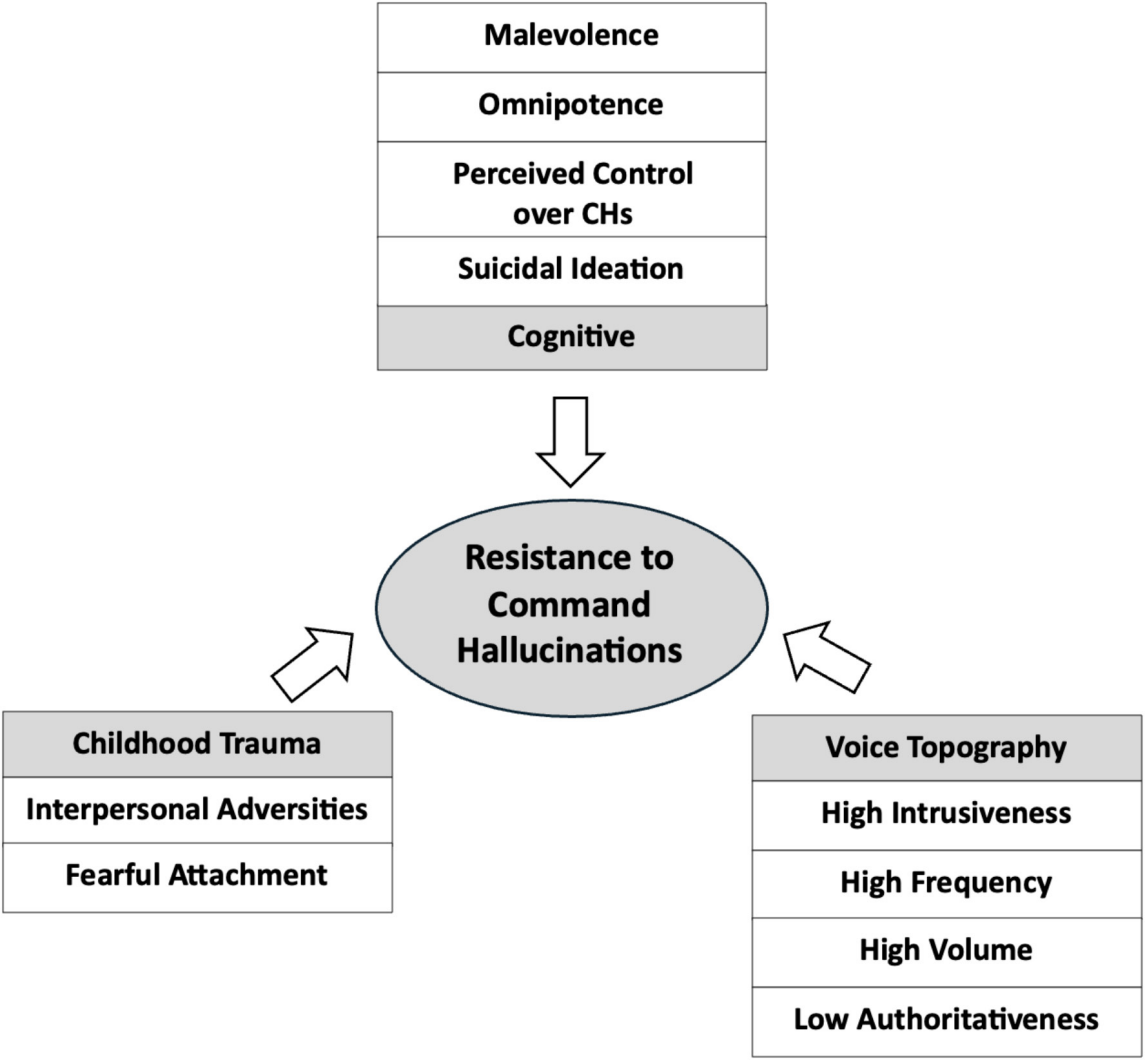
Factors associated with resistance to command hallucinations.

Based on the findings of this review, we have developed visual models summarising the clinical correlates of compliance, appeasement and resistance to CHs (see Figures 2, 3, 4, respectively). Compliance with CHs was associated with cognitive factors including beliefs about the voice's benevolence, omnipotence and omniscience, perceptions of the likelihood of future compliance, anticipated negative consequences of disobedience and greater attentive awareness. Relational factors, such as perceived social rank and characteristics of the voice (identity, familiarity), also contributed. Emotional factors—particularly anger and a compelling sense of obligation—combined with behavioural tendencies towards impulsivity and social isolation. Childhood trauma (physical, emotional abuse or neglect) and comorbid SUD were significant developmental and clinical contributors. Symptom severity, including hallucination‐related delusions, hospitalisation and medication non‐adherence, further influenced compliance behaviours.

Appeasement responses were characterised by cognitive appraisals of perceived dangerousness. Behavioural strategies aimed to reduce threat, such as avoiding provocation, complying with milder commands or engaging in self‐harm to prevent harm to others. These responses suggest that appeasement is primarily motivated by threat mitigation rather than obligation. As appeasement has been less extensively investigated than compliance, further research is needed to clarify the factors that specifically influence this response.

Resistance to CHs involved cognitive factors reflecting beliefs about the voice's malevolence and omnipotence, perceived control over the voice and concurrent suicidal ideation. Voice topography—high intrusiveness, high frequency, high volume and low authoritativeness—also influenced resistance. Trauma‐related factors, including childhood adversity, interpersonal adversities and a fearful attachment style, further shaped oppositional responses.

**FIGURE 5:**
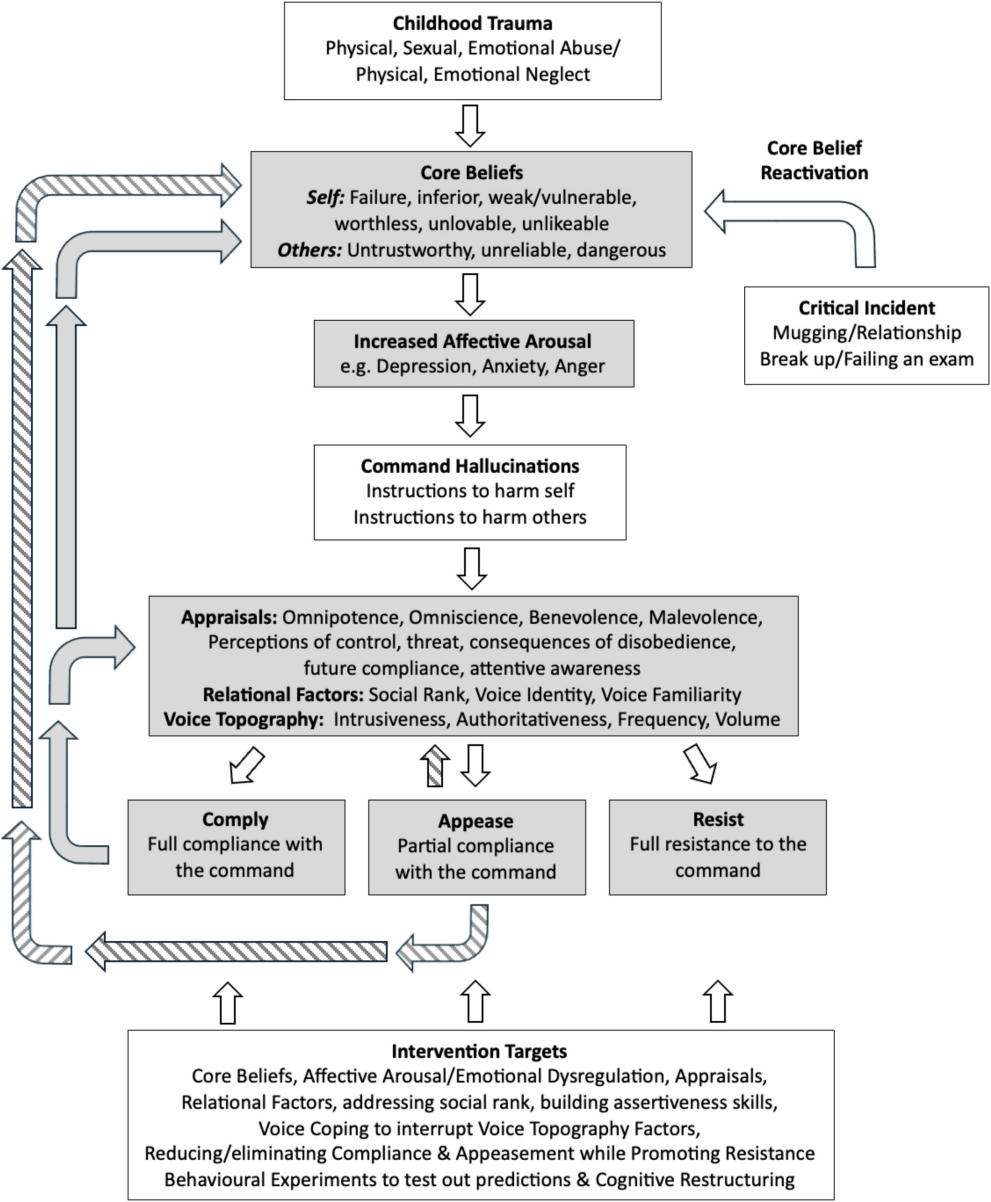
Cognitive model of compliance, appeasement and resistance to command hallucinations. *Note:* Clear arrows represent hypothesised directional pathways. Grey arrows indicate reinforcing feedback loops associated with compliance. Striped arrows represent partial or indirect reinforcement processes associated with appeasement.

Based on the findings of the present review, we propose a *Cognitive Model of Compliance, Appeasement, and Resistance to Command Hallucinations* (see Figure 5). This model posits that early life adversity, including childhood physical, sexual and emotional abuse, as well as physical and emotional neglect, contributes to the development of enduring negative core beliefs about the self (e.g., failure, inferior, weak/vulnerable, worthless, unlovable, unlikeable) and others (e.g., untrustworthy, unreliable, dangerous). These core beliefs confer a vulnerability for heightened threat sensitivity, emotional dysregulation and submissive interpersonal schemas, which are central to understanding responses to CHs. In addition to shaping enduring negative core beliefs, early interpersonal trauma may give rise to dissociative tendencies that disrupt self‐referential processing—the capacity to recognise thoughts, emotions and bodily states as originating from oneself. When early experiences are overwhelming or chronic, aspects of cognitive and emotional experience may become fragmented rather than fully integrated. Such dissociative tendencies increase the likelihood of intrusive thoughts or affective states being perceived as externally generated or independent of the self. In the context of CHs, difficulties in distinguishing between internally generated versus externally generated experiences may lead to a diminished sense of agency, whereby commands are experienced as arising from a powerful external source and not under voluntary control. Importantly, this reduced sense of agency may have paradoxical effects: perceiving the voice as externally generated may lead to compliance if it is appraised as powerful or threatening, but may elicit resistance if appraised as malevolent. Conversely, perceiving the voice as internally generated—and therefore self‐produced—may make it easier to dismiss or resist. Within the cognitive model, these effects are captured through appraisals, including perceptions of control, threat and anticipated consequences of disobedience, which shape subsequent behavioural responses. Taken together, these developmental and phenomenological processes provide a foundation for understanding how trauma‐related mechanisms influence voice‐hearing experiences and behavioural responses and inform the cognitive model of compliance, appeasement and resistance to CHs.

Critical incidents or psychosocial stressors serve to reactivate these pre‐existing core beliefs, leading to elevated affective arousal characterised by anxiety, sadness, anger, guilt or shame. Heightened affective arousal is proposed to play a key role in the emergence or intensification of anomalous experiences such as CHs (Jorovat et al. 2025; Gurnani and Georgiades 2025; Georgiades et al. 2023). The experience of CHs is not conceptualised as leading directly to behavioural responses; rather, responses are mediated by cognitive, relational and phenomenological appraisals of the voice. Specifically, individuals may form appraisals about the voice in terms of omnipotence, omniscience, benevolence or malevolence, perceived control, threat, anticipated consequences of disobedience and perceptions of future compliance. These beliefs interact with relational factors, such as the perceived social rank, identity and familiarity of the voice, as well as voice topography features such as intrusiveness, authoritativeness, frequency and volume. Together, these factors influence the perceived power and dominance of the voice in relation to the self and determine the degree of perceived agency available to the individual.

In this model, compliance, appeasement and resistance are understood as functionally distinct behavioural responses arising from these appraisals. Compliance and appeasement are hypothesised to be more likely when the voice is appraised as highly powerful, threatening and dominant and when the individual perceives themselves as weak, inferior or unable to cope with disobedience. In contrast, resistance is more likely when appraisals about the voice's power and authority are weakened, relational distance from the voice is increased and the individual's sense of agency and self‐efficacy is enhanced.

Crucially, compliance and appeasement are proposed to operate as maintaining mechanisms. Repeated compliance strengthens appraisals about the omnipotence and authority of the voice, increases perceived threat and reinforces submissive social rank appraisals. In turn, compliance also serves to confirm and entrench negative core beliefs about the self (e.g., ‘I am weak’ or ‘I am inferior’), thereby increasing vulnerability to future affective arousal and CHs. These feedback loops help to explain the persistence and escalation of harmful CHs over time. Resistance, by contrast, may weaken conviction in the appraisals of the voice's power and disrupt these maintaining cycles.

In the proposed model, appeasement is conceptualised as a functionally distinct yet maintaining response to CHs. Although appeasement involves partial or indirect obedience (e.g., avoiding provocation, complying with milder commands or engaging in self‐harm to reduce perceived threat), it is hypothesised to operate as a safety behaviour. By engaging in such behaviours, the individual perceives that they have mitigated the feared outcome or threat from occurring, alleviating distress, and thereby negatively reinforcing the appeasement behaviour. This negative reinforcement maintains appraisals of the voice as powerful, threatening and dominant and reinforces a submissive relational stance towards the voice. Over time, repeated appeasement is proposed to strengthen beliefs about the necessity of appeasing commands and reinforce negative core beliefs about the self (e.g., vulnerability or inferiority), thereby sustaining the cycle of CHs. Feedback loops from appeasement to both voice‐related appraisals and core beliefs are included, mirroring those observed for compliance, albeit potentially operating through more partial or ambiguous reinforcement mechanisms.

This model therefore highlights several key clinical intervention targets. These include modifying negative core beliefs, reducing affective arousal and emotional dysregulation and directly addressing beliefs about the voice and relational factors that confer perceived dominance, for example, by enhancing assertiveness skills. Interventions may also target voice topography through coping strategies designed to reduce intrusiveness and salience. A central therapeutic aim is to reduce compliance and appeasement while promoting resistance and assertive responding to voices. Behavioural experiments are proposed as a key mechanism for testing predictions about the consequences of disobedience, alongside cognitive restructuring to weaken beliefs about voice omnipotence and threat.

Overall, this model provides a cognitive–affective–relational framework for understanding differential responses to CHs and offers a clear rationale for targeted, individualised CBTp interventions aimed at reducing harm and increasing autonomy in individuals who experience commanding voices. To support clinical assessment and formulation, we have developed a set of Socratic questions that map onto the domains of compliance, appeasement and resistance to CHs (see Table 5). These questions are designed to facilitate structured exploration of beliefs, appraisals and behavioural responses to voices and to inform intervention strategies such as behavioural experiments and cognitive restructuring. Importantly, this Socratic question framework is intended to support the development of personalised and idiosyncratic formulations by explicitly operationalising the *Cognitive Model of Compliance, Appeasement, and Resistance to Command Hallucinations* within clinical practice. By mapping clients' responses onto specific components of the model, clinicians can collaboratively identify maintaining mechanisms and tailor interventions to target those processes most relevant to the individual.

**TABLE 5:** Socratic questions to assess for command hallucinations.

**Content** What does the voice say exactly? Is it a single word, phrase or sentence? Does the voice ever make personal comments about you? (e.g., that you are weak, vulnerable, inferior, worthless, unlovable or a failure?) **[core beliefs about self]** Does the voice ever make personal comments about others? (e.g., that they are untrustworthy, unreliable, critical or bad?) **[core beliefs about others]** Does the voice give you instructions or tell you to do things? (e.g., to move, to harm yourself or to harm others) How specific are the instructions the voice gives you, and does it say when or how to act? Does the voice ever say something like if you do not do X, then Y will happen? What does the voice say will happen if you disobey? Does the voice ever say anything positive? When the voice says something positive, how do you make sense of that? Does positive content change how much you trust or listen to the voice? What do you think the voice is trying to achieve by saying this? Do you think it is trying to help, protect, punish or control you?
**Triggers** What happens just before you start to hear the voice? (e.g., an argument, feeling lonely or sad, seeing something on the news or social media?) What thoughts do you have just before the voice starts? Are you thinking about anything in particular, such as worries, images or memories? What emotions do you tend to feel just before the voice starts? (e.g., sadness, anxiety, anger, guilt, shame or fear?) Were you experiencing any physical sensations just before the voice starts, such as tension, tiredness, hunger, illness or feeling emotionally overwhelmed? Is there a time of day when the voice is particularly active, or can it occur at any time? (e.g., upon waking, when falling asleep, during the day, evening or at night?) Is there anything in your physical environment that tends to trigger the voice? (e.g., loud noises, crowds, empty spaces, certain facial expressions, tone of voice, being stuck in traffic)? Does the voice start when you are alone or with someone? If it starts when you are with someone, what tends to happen in this social interaction? (e.g., did you feel safe/supported/criticised/judged or something else?) Do you tend to use alcohol and/or drugs before the voice starts? Or have you experienced any recent changes in your medication shortly before the voice starts? Does the voice tend to appear after reminders of stressful past events or traumatic experiences?
**Compliance** Do you ever fully comply with what the voice tells you to do? What does the voice say exactly, and how did you tend to interpret it? (e.g., the voice saying ‘there's a bridge’ means that the voice wants me to jump) How come? What makes you think the voice wants you to jump?—[client: Because the voice thinks I am bad and deserve punishment] Do you believe that you are bad? How do you view yourself when the voice says that?—[client: that I am worthless and a failure] **[Elicit core beliefs]** Which of these two beliefs about yourself upsets you the most? Worthlessness or failure? **[Identifying core belief that elicits the most distress]** Has there been a time in your past when you felt both worthless and like a failure? Can you describe what happened during that time? Do you notice any similarities between what the voice says and how you felt about yourself in the past? And what emotion do these beliefs bring up for you? (e.g., sadness, guilt and shame) And how do tend to respond to the voice? What do you think would happen if you did not comply with the voice? (e.g., that some harm will come to you or your family, that they will get louder/more intrusive/become more repetitive?) How convinced are you that this would actually happen? (0%–100% from not at all convinced to completely convinced?) Has the threat/feared prediction from the voice ever come true/ever happened? What helps you feel safer and more in control when the voice gives commands to harm yourself or others? What strategies, if any, have helped you to reduce compliance when the voice is active? Would you be willing to agree to delay or suspend compliance while we explore some alternative safety strategies? Could we explore strategies to help reduce the impact or intensity of the voice so you feel more in control? What happens to your anxiety or level of distress after you comply? (e.g., reduces, increases, stays the same?) Does compliance bring relief, even if only temporarily? And after some time has passed, what happens to that feeling of relief? Does it tend to stay the same, increase or decrease? How long does that relief last? **[captures negative reinforcement, a key maintenance mechanism]** Do you ever feel morally obligated to do what the voice says? Does the voice suggest you ‘should’ do this to be a good person, prevent harm or make up for something? Do you feel responsible if you do not obey? When did the voice first start giving commands? Was the first time you complied different from now? What did you do then and how do you tend to react now? Has the voice escalated its demands in any way over time?
**Appeasement** Do you ever partially comply with what the voice tells you to do (e.g., cutting yourself a little/superficially or walking near a bridge rather than doing something more serious), in order to appease the voice or reduce perceived threat? What does the voice ask you to do, and what do you actually do in response? (when you do not fully comply with the voice?) Have you ever engaged in behaviours to protect others from harm suggested by the voice? What happened? What thoughts or beliefs make you act on what the voice says? What do you think would happen if you did not carry out these behaviours? What leads you to partially comply with the voice command rather than fully comply? Have there been times when you mildly complied or delayed these behaviours? What was different in those moments? How do you feel before, during, and after engaging in these behaviours? Could there be other ways to manage the situation that does not involve self‐harm or appeasing the voice? What might it mean for the voice's power if you were able to act differently in these situations? What made you partially comply rather than completely resist?
**Resistance** Are there times when you are able to resist what the voice tells you to do? What helps you to resist the voice? What do you think would happen if you resisted the voice? Do you ever worry about any negative consequences if you were to resist the voice/not do what it tells you to do? (e.g., that something bad would happen? What would happen exactly? And how would you know the voice was the cause of it? E.g., if someone else is sick? What other possible explanations might explain that?) What kind of person do you want to be, regardless of what the voice says? When you are able to resist the voice, what is different about those moments? What factors or circumstances make it possible for you to resist the voice?
**Identity** Is the voice male or female? Is it human or a spiritual entity or something else? Do you have a sense regarding the identity of the voice? Is it familiar to you? Does it sound like anyone you know currently or from your past? Does the voice have good, bad, or neutral intentions towards you? **[assessing for benevolence vs. malevolence]**
**Omnipotence/Power** Who do you think is more powerful, you or the voice? How convinced are you out of 100 that the voice is powerful? (0% not at all convinced to 100% convinced) What does the voice tell you it will do to you? What do you think it could do? How could it do that? Has the voice ever done what you fear it could do? How do you know it could do this? Has the voice ever provided evidence for this power, or is it something it only claims?
**Omniscience/All Knowing** Do you believe the voice is all‐knowing and knows everything about you? What kinds of things do you think the voice knows about you? (e.g., your thoughts, intentions, past, future?) Does the voice ever claim to know what will happen in the future? Has it predicted any outcomes? Has the voice ever been wrong about something it claimed to know? Can you think of times when the voice did *not* seem to know what you were thinking or planning? When the voice appears to ‘know’ something accurately, how do you tend to explain it? Could there be another possible explanation for how the voice knows these things? (e.g., guessing, coincidence, the voice is actually your own disconnected thoughts that you no longer recognise) If the voice truly knew everything, what kinds of things should it be able to tell us? (e.g., what will be in the news tomorrow? What the exact temperature is in New York today? A passage from the bible selected at random?) Has the voice ever told you something verifiable that you did not already know? How could we gently test whether the voice knows things independently of your own thoughts or experiences? If the voice did *not* know everything, how powerful would it seem then? How much does the voice's power come from what it claims to know? Do you think the voice benefits from you believing it knows everything? What effect does this have on you?
**Behavioural Experiments** Collaboratively designed behavioural experiments to test beliefs about voice omnipotence (e.g., prediction testing unrelated to harm) If you have stopped going out of the house because the voice tells you to stay inside, what do you think would happen if you started to go out again? Is this something you would like to be able to do again? Could we test out your feared predictions together in a safe and gradual way to see whether the voice is as powerful as it says it is? How can we test how powerful the voice is? What ideas do you have for this? Could you imagine it is just a bluff, and the voice has no control or power over you at all, how would you feel?—[Client: I would feel silly]—maybe temporarily but is not it better to know now rather than lose any more time living in this restricted way because of the voice? How could we test how powerful the voice is? What if I picked a random page number and paragraph from a book, if the voice was truly powerful and all knowing, I bet it could tell me what the paragraph says? What do you think? What about if I write a word on a piece of paper, could it tell me what the word is? Or could it tell me what I ate for dinner yesterday?
**Purpose and Insight** Do you have a sense of why the voice is telling you these things? What is the purpose of the voice? (e.g., to punish you, protect you, warn you, motivate you, test you, to maintain control or something else?) Is the voice mental health related or due to a spiritual experience or something else? What is your understanding of why you might be hearing this voice? (e.g., related to any past traumas/too much stress/not enough sleep/something called psychosis or an intense spiritual experience?) Do you think the voice is mental health related or something entirely separate from you like a spiritual force or someone you knew in your past, e.g., a bully? If mental health related—Is it possible that due to difficult past experiences, you are now hearing a voice that is generated by a part of your brain that you no longer recognise as your own thoughts, so it feels separate from you? If the voice was generated by your own mind, how would that influence your perceptions of how powerful it is? [Client: The idea of the voice being powerful would completely disappear as it is down to my own brain, I would not be so scared] If externally attributed—If you understand the voice as spiritual, how do you make sense of it giving commands to harm yourself or others? How does this fit with your broader beliefs about that spiritual figure or force? Do you think God would tell you harm anyone? Is that consistent with religious teachings?
**Voice Topography** Do you hear the voice inside your head and/or outside your head? **[location]** Is it clear or muffled? **[clarity]** Is it the same volume as how we are speaking now or is it loud or a whisper? **[volume]** Do you notice changes in the voice's volume or intensity at different times of the day or in different situations? **[situational triggers]** How many voices do you hear? One or more? **[number of voices]** How often do you hear the voice? Once a day, every hour? Does it happen at specific times or all day? **[frequency]** What is the tone of the voice? Is it hostile, threatening, calm, positive or neutral? **[emotional valence of the voice]** How intrusive or distracting is the voice when it occurs? **[intrusiveness]** Does the voice ever feel overwhelming or impossible to ignore? **[degree of interference]** How authoritative or commanding does the voice feel? **[authoritativeness]** Are there times when the voice seems less controlling or less convincing? **[perceived control over the voice]** How do these characteristics (volume, frequency, intrusiveness, authoritativeness) affect how you respond to the voice? If the voice were less frequent, less loud or less authoritative, how would that change how you feel or act? Could there be any other explanations for why the voice feels so intrusive or powerful at times? (e.g., not sleeping enough so feel more vulnerable, low in mood so more likely to agree with the negative voice?) What strategies, if any, help you cope when the voice is very loud, frequent or intrusive? How much of the voice's perceived power do you think comes from how it presents itself (e.g., loud, frequent) versus what it says (e.g., content)? How much attention do you pay to the voice when it occurs? **[attentive awareness]** Are there times when you attend more to the voice? And times when you attend less to the voice? What seems to influence that?
**Coping** How do you tend to cope with the voices? What strategies do you tend to find helpful/unhelpful? Are there any strategies that make it better/worse/make no difference? Is there anything in your physical environment that tends to make it better or worse? Is there anyone in your social environment that tends to make it better or worse? What tends to happen? Does reading, counting backwards from 100 in 4's, listening to music or talking to someone help lessen the voice?
**Social Rank/Assertiveness** How do you tend to view yourself in relation to the voice—equal, superior or inferior? How do you tend to view the voice in terms of social standing—equal to you, superior to you or inferior to you? Do you feel you can stand up to the voice or be assertive with it? Would you be open to practising ways of being more assertive with the voice? Can we role play this together? Firstly, I'll role play the voice and you be you. Then you role play the voice and I will be assertive with the voice, then you try it. I can guide you with how to be more assertive with the voice so you can practice this in between our sessions. How might your beliefs about your social rank relative to the voice affect the way you respond to its commands? Are there times when you feel more equal or empowered in relation to the voice? What is different about those times?
**Childhood Trauma/Interpersonal Adversities** What are your early experiences of social interactions? With friends, family, strangers? Any bullying, exploitation, neglect, feeling unsafe or unsupported? How did your parents relate to each other? Verbally, physically and emotionally? How did you relate to each of your parents when you were a child? Attached/detached? Safe/fearful? How did you experience them? Warm/critical/hostile/unpredictable? How did you relate to your peers at school? How did your peers relate to you? How did these experiences shape how you view yourself? E.g., worthless and unlovable **[Core beliefs about self]** What about how you view others? E.g., superior and their needs more important than mine **[Core beliefs about others]** How do you think these early experiences might relate to how you respond to the voices now? When the voices give commands or criticisms, does this remind you of anyone from your past? When the voices are frightening or controlling, does it feel similar to how you have felt in your earlier relationships? Do you notice any connection between the way you feel about yourself and what the voices say? E.g., beliefs about your worth and lovability?
**Attachment Style/Relational Patterns** Did you find it easy or difficult to get close to people when you were growing up? How comfortable did you feel depending on others as a child? When you wanted support or comfort, did you feel able to ask for it? Did you worry about people leaving or rejecting you? How safe or unsafe did you feel showing your feelings to others/people you are supposed to trust (i.e., parents)? Looking back, do you notice any patterns in how you formed relationships as a child? Do you notice any patterns in your relationships now that seem similar to experiences from your childhood? When you experience commands or criticisms from voices, do they remind you of past relationships where you felt anxious, unsafe or dependent?

### 7.1 Comparison With Existing Cognitive and Relational Models of CHs

The proposed *Cognitive Model of Compliance, Appeasement, and Resistance to Command Hallucinations* builds upon and extends existing cognitive and relational models of voice‐hearing, particularly social rank and voice power frameworks, which emphasise the role of perceived voice omnipotence, dominance and threat in predicting compliance (Chadwick and Birchwood 1994). Consistent with these models, the present formulation highlights the central role of beliefs about voice power, authority, malevolence and benevolence, as well as relational appraisals such as social rank and submission, in influencing behavioural responses to voices. However, the current model extends prior accounts in several important ways. First, it explicitly embeds CHs within a broader developmental framework, linking early life adversity and trauma‐related core beliefs to later vulnerability for submissive interpersonal schemas and emotional dysregulation thereby emphasising an affective pathway to psychosis (Gurnani and Georgiades 2025). Second, rather than conceptualising behavioural responses as a direct consequence of voice power alone, the model proposes that responses are mediated by interacting cognitive, affective, relational and phenomenological appraisals of the voice, including voice topography and perceived agency. Third, the model maintains appeasement as a behavioural response that is conceptually and clinically distinct from both compliance and resistance. Appeasement behaviours (e.g., placating, negotiating, partial compliance or safety behaviours) function to reduce perceived threat without fully obeying or directly opposing the voice. While these behaviours may be experienced as protective, they can inadvertently increase risk by encouraging engagement in potentially harmful acts and reinforce beliefs about the voice's power and the individual's vulnerability, prolonging distress and limiting opportunities for disconfirmatory learning. Clinically, this distinction is important, as appeasement may be less readily identified than overt compliance or resistance, yet may play a significant role in maintaining voice‐related beliefs and sustaining maladaptive response patterns. Fourth, the inclusion of dynamic feedback loops illustrates how compliance and appeasement actively maintain both beliefs about voice power and negative core beliefs about the self, thereby accounting for the persistence and escalation of harmful CHs over time. Finally, the model delineates resistance not merely as the absence of compliance or appeasement, but as a clinically meaningful process that facilitates belief revision and increased agency, providing a clear rationale for intervention strategies such as assertiveness training, behavioural experiments and cognitive restructuring within CBT for psychosis. In doing so, the proposed model offers an integrative and formulation‐driven framework that complements and extends existing social rank approaches by explicitly linking developmental vulnerability, appraisal processes and mechanisms of change.

Comparative insights reveal both shared and distinct factors across response types. Beliefs about omnipotence were present in both compliance and resistance, highlighting the centrality of perceived voice power. Malevolence was associated with resistance, whereas benevolence was unique to compliance, suggesting elevated clinical risk when clients attribute positive intentions to voices. However, the role of omnipotence and evaluative beliefs about malevolence and benevolence in appeasement remains unclear. Emotional and behavioural patterns further differentiated responses: compliance was marked by feelings of obligation and submission, appeasement by threat‐reducing strategies and resistance by oppositional or assertive behaviours. Trauma and relational factors modulated both compliance and resistance, indicating the influence of early experiences on coping strategies.

Social rank appears to be a critical relational determinant of responses to CHs. Individuals who perceive themselves as subordinate to the voice are more likely to comply with commands, particularly when commands involve self‐harm (Fox et al. 2004; Barrowcliff and Haddock 2010). Conversely, partial compliance or appeasement may emerge when individuals perceive the voice as powerful but anticipate harmful consequences from full compliance (Byrne et al. 2003). Social rank theory suggests that one's perceived position within social hierarchies influences voice appraisals, such that those with low self‐perceived social rank are more likely to attribute omnipotence to the voice and engage in submissive behaviours (Paulik 2012; Griffiths et al. 2012).

Furthermore, how individuals relate to their voices appears to mirror how they relate to others in their social relationships (Griffiths et al. 2012; Hayward 2003). Those who perceive themselves as inferior in social contexts may similarly perceive themselves as subordinate to their voices, reinforcing compliance and appeasement. This suggests a possible avenue for intervention: developing assertiveness skills in interpersonal relationships could generalise to interactions with their voice, helping to recalibrate perceived power imbalances in the client‐voice relationship (Craig et al. 2018; Hayward et al. 2017). Clinically, assessing social rank perceptions and targeting both social and voice‐related assertiveness may reduce maladaptive compliance and support more adaptive coping.

Attachment patterns also appear to shape responses to CHs by influencing interpretations of voice intent and power. Individuals with avoidant or fearful attachment styles were found to appraise voices as malevolent, dominant or threatening, increasing resistance or oppositional behaviours (Berry et al. 2012; Robson and Mason 2015). In contrast, those with secure attachment may experience less distress and greater flexibility in coping strategies. However, it is important to note that a limited number of studies explored the direct role of trauma subtypes in relation to compliance, appeasement and resistance, so it is unclear how attachment style influences these three response styles to CHs. Nevertheless, a consideration of attachment style in clinical assessments may provide insight into the likelihood of resisting CHs.

Childhood trauma was found to contribute to both compliance and resistance. However, the mechanisms underlying this relationship remain underexplored. Core beliefs may be a potential cognitive mediator influencing the relationship between trauma and behavioural responses to CHs. For example, childhood abuse and neglect may lead to the formation of core beliefs concerning weakness/vulnerability, inferiority, unlovability, worthlessness and failure, contributing to feelings of lower social rank and increasing the likelihood of subsequent compliance with commands (Çelik and Odacı 2012; Cruz et al. 2022). This is supported by findings that childhood abuse is a significant predictor of submissive behaviour (Çelik and Odacı 2012). Therefore, individuals who have experienced trauma and comply with commands may benefit from interventions not only focusing on improving one's own social rank but also addressing negative core beliefs about the self and enhancing more positive self‐beliefs (Jorovat et al. 2025).

Spiritual and religious beliefs may also influence appraisals and behavioural responses to CHs (Ghanem et al., Westhead and Georgiades 2025). Voices attributed to culturally or spiritually significant entities—such as God, ancestors or family members—are more likely to elicit compliance, potentially through trust or reverence, whereas voices identified as demonic or authoritative figures may evoke resistance (Luhrmann et al. 2015; Beck‐Sander et al. 1997; Ghanem et al. 2023). These findings highlight the importance of exploring clients' cultural and spiritual frameworks during the assessment and formulation of CHs, as these beliefs may modulate both risk and coping behaviours. Clinicians could explore how spiritual appraisals intersect with cognitive appraisals of omnipotence, benevolence or malevolence when devising CBTp formulations and planning targeted interventions.

Negative voice content is a well‐established predictor of distress and functional impairment, often necessitating contact with mental health services (Larøi et al. 2019). However, its specific role in shaping responses to CHs has been explored primarily in relation to compliance. It remains unclear how negative voice content influences appeasement or resistance behaviours, limiting our understanding of the mechanisms driving these response patterns. Given the potential for voice content to affect perceived threat, emotional responses and behavioural strategies, further research examining its impact across all response types is warranted.

Higher symptom severity has been consistently associated with increased compliance to CHs. Individuals with more pronounced positive and negative symptoms of psychosis, hallucination‐related delusions, non‐adherence to antipsychotic medication or longer hospitalisation periods are more likely to comply with commands (Junginger 1990; Salim et al. 2021; Shawyer et al. 2008). Greater symptom severity may impair the individual's ability to accurately appraise the reality and intent of voices, leading to stronger beliefs in the voices' omnipotence and reinforcing compliance (Salim et al. 2021). Clinically, assessing symptom severity is crucial for formulation development, and interventions targeting reality testing, symptom management and psychoeducation may help reduce maladaptive compliance by challenging the perceived power and credibility of voices.

Comorbid SUDs have also been associated with increased compliance with CHs (Dugré et al. 2018). SUDs can impair inhibitory control and decision‐making, making it more difficult for individuals to evaluate whether compliance or resistance is adaptive (Dugré et al. 2018; Murphy et al. 2012). Additionally, individuals with psychosis and comorbid substance use often exhibit elevated impulsivity, which further increases the likelihood of complying with commands (Duva et al. 2011; Bucci et al. 2013). Clinically, assessing substance use is essential, as interventions targeting SUDs—such as harm reduction strategies, motivational interviewing and cognitive‐behavioural approaches—may indirectly reduce compliance by improving cognitive control and decision‐making capacity.

Voice topography—such as intrusiveness, authoritativeness, frequency and loudness—can influence responses to CHs. Resistance is more likely when voices are highly intrusive, frequent or loud and perceived as less authoritative (Andrew et al. 2008; Mackinnon et al. 2004; Soppitt and Birchwood 1997). Louder voices were also associated with malevolent appraisals, potentially increasing oppositional behaviours. While some evidence suggests that cognitive appraisals may be more influential than voice activity alone (Birchwood and Chadwick 1997), topography remains an important consideration in assessment and formulation. Understanding these characteristics can also inform interventions via coping strategies aimed at reducing the impact of distressing or intrusive voices, helping individuals manage the influence of topographical features on behavioural responses.

Clinical implications include the importance of assessing clients' appraisals of voice intent, perceived power and anticipated consequences of disobedience. Interventions such as Socratic questioning and behavioural experiments can enhance perceived control over voices, challenge maladaptive beliefs and reduce risk behaviours. For example, testing feared outcomes of noncompliance in a controlled manner may help clients gain confidence and reduce compulsive appeasement or compliance behaviours. Targeting impulsivity, social isolation and perceived threat also represents meaningful avenues for intervention.

In summary, these findings provide a comprehensive framework for understanding the clinical correlates of responses to CHs, offering actionable insights for clinical assessment, formulation and intervention—particularly within CBTp approaches. They underscore the importance of individualised strategies based on clients' unique appraisals, behaviour and relational context.

### 7.2 Strengths and Limitations

This systematic review sought to investigate the clinical correlates of compliance, appeasement and resistance in CHs. Including both quantitative and qualitative studies enabled a comprehensive synthesis of the extant findings, which is a strength of the current review (Sandelowski et al. 2006). This approach supported the development of visual summaries outlining what is currently known about these response domains and identifying areas that remain under‐investigated, supporting the development of future research into CHs. In addition, the review explored specific command typologies—such as self‐harm and harm‐to‐others commands—thereby enhancing understanding of how clinical correlates may differ across distinct forms of CHs.

Some limitations should be noted. Despite conducting both database and manual searches, some relevant papers may not have been captured. Restricting inclusion to English‐language, peer‐reviewed publications also risked omitting insights from grey literature or non‐English sources. Furthermore, the predominance of cross‐sectional designs limits causal inferences from being made regarding relationships between clinical variables and responses to CHs. Moreover, the samples across the included studies had a high mean duration of illness (7.4–28.87 years), representing more chronic psychosis presentations. These findings are therefore not generalisable to early psychosis samples, whose responses and coping strategies may differ.

There was marked variation in how compliance‐related responses to CHs were operationalised across studies. Some combined partial and full compliance due to small sample sizes (Barrowcliff and Haddock 2010) or combined compliance and appeasement under the broader category of safety behaviours (Hacker et al. 2008), while others defined compliance dichotomously as the presence of any partial or full compliance with at least one severe command (Birchwood and Trower 2006; Shawyer et al. 2007; Salim et al. 2021). In contrast, two studies combined appeasement with resistance behaviours, further contributing to inconsistency in outcome definitions (Beck‐Sander et al. 1997; Reynolds and Scragg 2010). Such practices may obscure potentially distinct clinical correlates associated with different response types and risk underestimating the significance of appeasement, a clinically important construct that may carry elevated risk through inadvertent harm. Appeasement also remains comparatively underexplored relative to compliance and resistance.

The evidence base is additionally constrained by limited cultural diversity, with most studies being conducted in Western contexts (*n* = 49), particularly the United Kingdom (*n* = 28), with only seven studies from non‐Western settings. Cultural and spiritual frameworks may shape the meaning and response to CHs, underscoring the need for research exploring the cultural and spiritual influences on coping with CHs (Ghanem et al. 2023; Westhead and Georgiades 2025). Substance use is another potential confounding factor. Although comorbid SUDs have been associated with increased compliance (Dugré et al. 2018), most studies did not systematically assess or control for current substance misuse.

Finally, there was inconsistency in the range of correlates examined across studies. Some factors—such as attachment patterns, voice topography and core beliefs or trauma‐related mechanisms—were explored in only a small number of studies. Longitudinal research is therefore needed to clarify causal pathways between trauma, appraisals and behavioural responses to CHs and to build a more coherent, clinically actionable evidence base.

### 7.3 Clinical Implications

The findings of the present review highlight several important clinical implications for the assessment and treatment of CHs. First, the results highlight the need for explicit assessment of the full range of clinical correlates associated with compliance, appeasement and resistance. These factors—including trauma history, core beliefs, cognitive appraisals, emotional responses, voice characteristics, identity‐related beliefs and contextual triggers—should be incorporated into personalised case formulations. Doing so enables clinicians to develop *targeted*, mechanism‐based interventions within CBTp.

A key implication of this review is the clinical importance of recognising and assessing appeasement, a response style that is often overlooked or minimised. Although appeasement is sometimes conceptualised as involving relatively innocuous acts, evidence indicates that it can involve dangerous or self‐harming behaviours, often driven by a desire to placate the voice. As such, appeasement should be treated as a risk‐relevant response domain rather than a benign or low‐priority presentation. Routine enquiry into whether individuals self‐harm—or engage in risk‐taking behaviour—to appease voices should be standard practice in risk assessment.

This review also underscores the importance of reducing and eliminating unhelpful responses such as compliance and appeasement, while simultaneously strengthening factors that support safe and adaptive resistance. Traditional CBTp for CHs targets beliefs about voice omnipotence, which can reduce compliance (Birchwood and Trower 2006; Pontillo et al. 2016). However, the current review suggests that clinicians should broaden their focus. For example, benevolent beliefs about the voice—often perceived as less clinically concerning—were found to be associated with compliance. Targeting these beliefs may therefore reduce motivation to comply, challenge assumptions that the voice is helpful or protective and reduce risk.

For individuals who already resist, clinical work should aim to consolidate resistance while addressing the distress commonly associated with malevolent appraisals, loud voices and threatening content. Interventions such as coping strategy enhancement, grounding techniques, behavioural activation and supportive engagement (e.g., befriending) (Hayward et al. 2018; Shawyer et al. 2012) can help manage distress without inadvertently encouraging compliance.

Another important implication concerns the role of culture and spirituality. These frameworks often shape how voices are interpreted, the perceived legitimacy of commands and the meaning attached to compliance, appeasement or resistance. Clinicians should therefore explore cultural and spiritual contexts during assessment and formulation and integrate these considerations into culturally attuned CBTp interventions. This enhances therapeutic relevance and reduces the risk of pathologising beliefs that are normative within the client's cultural or spiritual framework.

Finally, as part of this review, a set of Socratic questions was developed to support clinicians in assessing CHs and exploring the cognitive, emotional and contextual factors underlying different response styles. We also propose the *Cognitive Model of Compliance, Appeasement, and Resistance to Command Hallucinations* to enhance personalised formulation development within CBTp. Together, these Socratic questions and cognitive model may facilitate more nuanced clinical conversations, improve formulation accuracy and support the delivery of personalised and effective interventions.

### 7.4 Future Directions

Future research should employ larger samples of individuals who engage in compliance, appeasement and resistance in order to delineate the clinical correlates unique to each behavioural response. Given that the existing evidence base was characterised by chronic psychosis presentations, it is important to examine CHs within early psychosis populations, including individuals with first episode psychosis (FEP). Comparing early‐stage and chronic presentations would clarify whether responses to CHs differ across illness stages, offering insights into initial coping styles and informing the development of stage‐specific assessment and intervention strategies.

Trauma has been implicated in the emergence of CHs, yet the mechanisms underpinning the association between childhood trauma subtypes and specific response types remain underexplored. An exploration of cognitive mediators such as core beliefs might help to explain the relationship between trauma and CHs (Jorovat et al. 2025). Specifically, it might help explain how trauma shapes behavioural reactions to CHs and could identify intervention targets for disrupting maladaptive pathways (Jorovat et al. 2025).

Emerging evidence highlights the importance of tailoring interventions for CHs based on trauma history and subtype. Voices arising from posttraumatic stress reactions, such as intrusions or dissociation following multiple trauma exposures, may respond more effectively to trauma‐focused therapies, whereas individuals with low trauma but persisting voices may benefit more from traditional CBT for psychosis (Brand et al. 2021; Lincoln and Peters 2019; Begemann et al. 2022). Examining differential treatment responses across trauma subtypes could help optimise interventions and ensure that treatment targets mechanisms most relevant to each individual's experience (Begemann et al. 2022).

Particular attention should also be given to voice appraisals, including omnipotence, which appears across both compliance and resistance. Voice topography (e.g., loudness, intrusiveness, frequency and identity) remains relatively under‐investigated in relation to compliance, appeasement and resistance, and it is unclear which specific features influence different behavioural responses. Broader under‐investigated factors—including cultural and spiritual frameworks, social support, interpersonal dynamics and medication adherence—should also be examined, and direct comparisons of response types within the same sample remain rare. Overall, the interplay between voice characteristics, cognitive appraisals, social context and situational triggers warrants further investigation.

Longitudinal studies represent a critical gap in the evidence base. Little is known about how responses to CHs unfold over time, whether individuals maintain a stable coping strategy or if they shift between strategies (e.g., from compliance to appeasement or to resistance) in response to changes in symptomatology, environment or treatment. Mapping these temporal patterns—from the onset of psychosis through several years post‐onset—would provide essential information for designing interventions that promote safer and more adaptive coping.

Ecological Momentary Assessment (EMA) offers a promising methodology for capturing real‐time fluctuations in urges to comply, appease or resist. Only two EMA studies in CHs have been conducted to date and exclusively in chronic psychosis (Fielding‐Smith et al. 2022; So et al. 2021). EMA not only allows measurement of moment‐to‐moment response behaviours but also enables examination of dynamic emotional and cognitive processes. For example, emerging evidence suggests that negative affect (NA) and AVH mutually reinforce each other in a feedback loop (So et al. 2021). This association is exacerbated in individuals who hold beliefs that voices are malevolent or omnipotent, as such beliefs increase perceived threat and helplessness, amplifying NA in response to voice experiences (So et al. 2021). Elevated NA may, in turn, increase susceptibility to distressing voices and influence how individuals respond to commands (So et al. 2021). Importantly, this finding is consistent with the Affective Pathway Model of Psychosis, which posits that escalations in NA contribute to the emergence of psychosis onset and the persistence of symptoms (Gurnani and Georgiades 2025). Targeting NA through emotional regulation strategies could help disrupt this loop, reducing AVH frequency and the likelihood of compliance or appeasement while supporting resistance (So et al. 2021). Future EMA research could clarify the moment‐to‐moment emotional, cognitive and contextual factors that precipitate risk‐related behaviours, thereby identifying precise therapeutic targets for CBTp.

There is also a notable lack of qualitative research exploring the clients' perspective of the factors influencing compliance, appeasement and resistance to CHs. Gaining insight into the subjective experience of coping in response to CHs would offer clinically meaningful data that could enhance the personal relevance and effectiveness of CBTp interventions.

### 7.5 Conclusion

This systematic review synthesised the clinical correlates associated with compliance, appeasement and resistance to CHs in psychosis. Compliance was associated with *cognitive factors* (benevolence, omnipotence and omniscience beliefs, perceptions of future compliance, perceived consequences of disobedience, greater attentional awareness), *relational factors* (social rank, voice identity, voice familiarity), *emotional drivers* (anger, obligation), *behaviours* (impulsivity, social isolation), *childhood trauma*, *substance use* and *overall symptom severity*. Appeasement was associated with *cognitive factors* (perceived dangerousness) and *behaviours* (avoiding provocation, obeying milder commands or self‐harm to protect others). Resistance was associated with *cognitive factors* (malevolence and omnipotence beliefs, perceived control over the voice, concurrent suicidal ideation), *voice topography factors* (high intrusiveness/frequency/volume and low authoritativeness), alongside *childhood trauma factors* (interpersonal adversities, fearful attachment). These findings highlight the need for clinical formulations of CHs to attend closely to the factors driving compliance and appeasement, given their strong association with risk. Targeting the cognitive, relational, emotional, behavioural and developmental factors that sustain these responses—and strengthening resistance‐promoting mechanisms—is therefore of clinical importance. The proposed Socratic questions and *Cognitive Model of Compliance, Appeasement, and Resistance to Command Hallucinations* provide a framework for comprehensive assessments, personalised formulations and targeted interventions in CBTp, thereby supporting the recovery of individuals experiencing CHs.

## Funding

The authors have nothing to report.

## Ethics Statement

The authors have abided by the Ethical Principles of Psychologists and Code of Conduct as set out by the BABCP and BPS.

## Conflicts of Interest

The authors declare no conflicts of interest.

## Data Availability

Data sharing is not applicable to this article as no new data were created or analysed in this study.

## References

[cpp70246-bib-0001] American Psychiatric Association . 2013. Diagnostic and Statistical Manual of Mental Disorders. 5th ed. American Psychiatric Association. 10.1176/appi.books.9780890425596.

[cpp70246-bib-0002] Andrew, E. M. , N. S. Gray , and R. J. Snowden . 2008. “The Relationship Between Trauma and Beliefs About Hearing Voices: A Study of Psychiatric and Non‐Psychiatric Voice Hearers.” Psychological Medicine 38, no. 10: 1409–1417. 10.1017/S003329170700253X. 18177529

[cpp70246-bib-0003] Barrowcliff, A. L. , and G. Haddock . 2010. “Factors Affecting Compliance and Resistance to Auditory Command Hallucinations: Perceptions of a Clinical Population.” Journal of Mental Health (Abingdon, England) 19, no. 6: 542–552. 10.3109/09638237.2010.520365. 20874508

[cpp70246-bib-0004] Beck‐Sander, A. , M. Birchwood , and P. Chadwick . 1997. “Acting on Command Hallucinations: A Cognitive Approach.” British Journal of Clinical Psychology 36, no. 1: 139–148. 10.1111/j.2044-8260.1997.tb01237.x. 9051285

[cpp70246-bib-0005] Begemann, M. J. H. , I. E. Sommer , R. M. Brand , et al. 2022. “Auditory Verbal Hallucinations and Childhood Trauma Subtypes Across the Psychosis Continuum: A Cluster Analysis.” Cognitive Neuropsychiatry 27, no. 2–3: 150–168. 10.1080/13546805.2021.1925235. 33980128

[cpp70246-bib-0006] Berry, K. , A. Wearden , C. Barrowclough , L. Oakland , and J. Bradley . 2012. “An Investigation of Adult Attachment and the Nature of Relationships With Voices.” British Journal of Clinical Psychology 51, no. 3: 280–291. 10.1111/j.2044-8260.2011.02027.x. 22803935

[cpp70246-bib-0106] Birchwood, M. 2003. “Pathways to Emotional Dysfunction in First‐Episode Psychosis.” British Journal of Psychiatry: The Journal of Mental Science 182: 373–375. 12724236

[cpp70246-bib-0007] Birchwood, M. , and P. Chadwick . 1997. “The Omnipotence of Voices: Testing the Validity of a Cognitive Model.” Psychological Medicine 27, no. 6: 1345–1353. 10.1017/s0033291797005552. 9403906

[cpp70246-bib-0008] Birchwood, M. , G. Dunn , A. Meaden , et al. 2018. “The COMMAND Trial of Cognitive Therapy to Prevent Harmful Compliance With Command Hallucinations: Predictors of Outcome and Mediators of Change.” Psychological Medicine 48, no. 12: 1966–1974. 10.1017/S0033291717003488. 29202885 PMC6137373

[cpp70246-bib-0009] Birchwood, M. , P. Gilbert , J. Gilbert , et al. 2004. “Interpersonal and Role‐Related Schema Influence the Relationship With the Dominant ‘Voice’ in Schizophrenia: A Comparison of Three Models.” Psychological Medicine 34, no. 8: 1571–1580. 10.1017/s0033291704002636. 15724887

[cpp70246-bib-0010] Birchwood, M. , A. Meaden , P. Trower , P. Gilbert , and J. Plaistow . 2000. “The Power and Omnipotence of Voices: Subordination and Entrapment by Voices and Significant Others.” Psychological Medicine 30, no. 2: 337–344. 10.1017/s0033291799001828. 10824654

[cpp70246-bib-0011] Birchwood, M. , M. Michail , A. Meaden , et al. 2014. “Cognitive Behaviour Therapy to Prevent Harmful Compliance With Command Hallucinations (COMMAND): A Randomised Controlled Trial.” Lancet Psychiatry 1, no. 1: 23–33. 10.1016/S2215-0366(14)70247-0. 26360400

[cpp70246-bib-0012] Birchwood, M. , and P. Trower . 2006. “Cognitive Therapy for Command Hallucinations: Not a Quasi‐Neuroleptic.” Journal of Contemporary Psychotherapy: On the Cutting Edge of Modern Developments in Psychotherapy 36, no. 1: 1–7. 10.1007/s10879-005-9000-y.

[cpp70246-bib-0013] Braham, L. G. , P. Trower , and M. Birchwood . 2004. “Acting on Command Hallucinations and Dangerous Behavior: A Critique of the Major Findings in the Last Decade.” Clinical Psychology Review 24, no. 5: 513–528. 10.1016/j.cpr.2004.04.002. 15325743

[cpp70246-bib-0014] Brand, R. M. , S. Bendall , A. Hardy , S. L. Rossell , and N. Thomas . 2021. “Trauma‐Focused Imaginal Exposure for Auditory Hallucinations: A Case Series.” Psychology and Psychotherapy 94 Suppl 2, no. Suppl 2: 408–425. 10.1111/papt.12284. 32436342 PMC8246845

[cpp70246-bib-0116] Buccheri, R. , L. Trygstad , and G. Dowling . 2007. “Behavioral Management of Command Hallucinations to Harm in Schizophrenia.” Journal of Psychosocial Nursing and Mental Health Services 45, no. 9: 46–54. 10.3928/02793695-20070901-11. 17907687

[cpp70246-bib-0015] Bucci, S. , M. Birchwood , L. Twist , N. Tarrier , R. Emsley , and G. Haddock . 2013. “Predicting Compliance With Command Hallucinations: Anger, Impulsivity and Appraisals of Voices' Power and Intent.” Schizophrenia Research 147, no. 1: 163–168. 10.1016/j.schres.2013.02.037. 23537476

[cpp70246-bib-0114] Byrne, S. , M. Birchwood , P. Trower , and A. Meaden . 2006. A Casebook for Cognitive Behaviour Therapy for Command Hallucinations: A Social Rank Theory Approach. Routledge.

[cpp70246-bib-0016] Byrne, S. , P. Trower , M. Birchwood , A. Meaden , and A. Nelson . 2003. “Command Hallucinations: Cognitive Theory, Therapy, and Research.” Journal of Cognitive Psychotherapy 17, no. 1: 67–84. 10.1891/jcop.17.1.67.58271.

[cpp70246-bib-0017] Çelik, Ç. B. , and H. Odacı . 2012. “The Effect of Experience of Childhood Abuse Among University Students on Self‐Perception and Submissive Behavior.” Children and Youth Services Review 34, no. 1: 200–204. 10.1016/j.childyouth.2011.09.017.

[cpp70246-bib-0018] Chadwick, P. , E. Barnbrook , and K. Newman‐Taylor . 2007. “Responding Mindfully to Distressing Voices: Links With Meaning, Affect and Relationship With Voice.” Tidsskrift for Norsk Psykologforening 44, no. 5: 581–587.

[cpp70246-bib-0019] Chadwick, P. , and M. Birchwood . 1994. “The Omnipotence of Voices. A Cognitive Approach to Auditory Hallucinations.” British Journal of Psychiatry: The Journal of Mental Science 164, no. 2: 190–201. 10.1192/bjp.164.2.190. 8173822

[cpp70246-bib-0020] Chadwick, P. , and M. Birchwood . 1995. “The Omnipotence of Voices. II: The Beliefs About Voices Questionnaire (BAVQ).” British Journal of Psychiatry: The Journal of Mental Science 166, no. 6: 773–776. 10.1192/bjp.166.6.773. 7663826

[cpp70246-bib-0021] Chadwick, P. , S. Lees , and M. Birchwood . 2000. “The Revised Beliefs About Voices Questionnaire (BAVQ‐R).” British Journal of Psychiatry 177: 229–232. 10.1192/bjp.177.3.229. 11040883

[cpp70246-bib-0113] Chadwick, P. , S. Sambrooke , S. Rasch , and E. Davies . 2000. “Challenging the Omnipotence of Voices: Group Cognitive Behavior Therapy for Voices.” Behaviour Research and Therapy 38: 993–1003. 11004738 10.1016/s0005-7967(99)00126-6

[cpp70246-bib-0022] Chaix, J. , E. Ma , A. Nguyen , M. A. Ortiz Collado , S. Rexhaj , and J. Favrod . 2014. “Safety‐Seeking Behaviours and Verbal Auditory Hallucinations in Schizophrenia.” Psychiatry Research 220, no. 1–2: 158–162. 10.1016/j.psychres.2014.08.041. 25219615

[cpp70246-bib-0023] Chawla, N. , R. Deep , S. K. Khandelwal , and A. Garg . 2019. “Beliefs About Voices and Their Relation to Severity of Psychosis in Chronic Schizophrenia Patients.” Indian Journal of Psychiatry 61, no. 5: 465–471. 10.4103/psychiatry.IndianJPsychiatry_573_18. 31579183 PMC6767829

[cpp70246-bib-0024] Close, H. , and P. Garety . 1998. “Cognitive Assessment of Voices: Further Developments in Understanding the Emotional Impact of Voices.” British Journal of Clinical Psychology 37, no. 2: 173–188. 10.1111/j.2044-8260.1998.tb01292.x. 9631205

[cpp70246-bib-0025] Craig, T. K. , M. Rus‐Calafell , T. Ward , et al. 2018. “AVATAR Therapy for Auditory Verbal Hallucinations in People With Psychosis: A Single‐Blind, Randomised Controlled Trial.” Lancet. Psychiatry 5, no. 1: 31–40. 10.1016/S2215-0366(17)30427-3. 29175276 PMC5746597

[cpp70246-bib-0026] Cruz, D. , M. Lichten , K. Berg , and P. George . 2022. “Developmental Trauma: Conceptual Framework, Associated Risks and Comorbidities, and Evaluation and Treatment.” Frontiers in Psychiatry 13: 800687. 10.3389/fpsyt.2022.800687. 35935425 PMC9352895

[cpp70246-bib-0119] Csipke, E. , and P. J. Kinderman . 2006. “A Longitudinal Investigation of Beliefs About Voices.” Behavioural and Cognitive Psychotherapy 34: 365–369.

[cpp70246-bib-0027] Denno, P. , S. Wallis , K. Caldwell , et al. 2022. “Listening to Voices: Understanding and Self‐Management of Auditory Verbal Hallucinations in Young Adults.” Psychosis: Psychological, Social and Integrative Approaches 14, no. 3: 281–292. 10.1080/17522439.2021.1964583.

[cpp70246-bib-0118] Drake, R. E. , and P. G. Cotton . 1986. “Depression, Hopelessness and Suicide in Chronic Schizophrenia.” British Journal of Psychiatry: The Journal of Mental Science 148: 554–559. 10.1192/bjp.148.5.554. 3779226

[cpp70246-bib-0028] Dugré, J. R. , J. P. Guay , and A. Dumais . 2018. “Risk Factors of Compliance With Self‐Harm Command Hallucinations in Individuals With Affective and Non‐Affective Psychosis.” Schizophrenia Research 195: 115–121. 10.1016/j.schres.2017.09.001. 28911915

[cpp70246-bib-0029] Dugré, J. R. , and M. L. West . 2019. “Disentangling Compliance With Command Hallucinations: Heterogeneity of Voice Intents and Their Clinical Correlates.” Schizophrenia Research 212: 33–39. 10.1016/j.schres.2019.08.016. 31451299

[cpp70246-bib-0030] Duva, S. M. , S. M. Silverstein , and R. Spiga . 2011. “Impulsivity and Risk‐Taking in Co‐Occurring Psychotic Disorders and Substance Abuse.” Psychiatry Research 186, no. 2–3: 351–355. 20870294 10.1016/j.psychres.2010.08.014

[cpp70246-bib-0111] Ehring, T. , R. Welboren , N. Morina , J. M. Wicherts , J. Freitag , and P. M. Emmelkamp . 2014. “Meta‐Analysis of Psychological Treatments for Posttraumatic Stress Disorder in Adult Survivors of Childhood Abuse.” Clinical Psychology Review 34, no. 8: 645–657. 25455628 10.1016/j.cpr.2014.10.004

[cpp70246-bib-0031] Ellett, L. , O. Luzon , M. Birchwood , Z. Abbas , A. Harris , and P. Chadwick . 2017. “Distress, Omnipotence, and Responsibility Beliefs in Command Hallucinations.” British Journal of Clinical Psychology 56, no. 3: 303–309. 10.1111/bjc.12139. 28493561

[cpp70246-bib-0032] Erkwoh, R. , K. Willmes , A. Eming‐Erdmann , and H. J. Kunert . 2002. “Command Hallucinations: Who Obeys and Who Resists When?” Psychopathology 35, no. 5: 272–279. 10.1159/000067065. 12457018

[cpp70246-bib-0033] Favrod, J. , F. Grasset , S. Spreng , B. Grossenbacher , and Y. Hodé . 2004. “Benevolent Voices Are Not so Kind: The Functional Significance of Auditory Hallucinations.” Psychopathology 37, no. 6: 304–308. 10.1159/000082269. 15564791

[cpp70246-bib-0034] Fenekou, V. , and E. Georgaca . 2009. “Exploring the Experience of Hearing Voices: A Qualitative Study.” Psychosis 2, no. 2: 134–143. 10.1080/17522430903191783.

[cpp70246-bib-0035] Fielding‐Smith, S. F. , K. E. Greenwood , M. Wichers , E. Peters , and M. Hayward . 2022. “Associations Between Responses to Voices, Distress and Appraisals During Daily Life: An Ecological Validation of the Cognitive Behavioural Model.” Psychological Medicine 52, no. 3: 538–547. 10.1017/S0033291720002238. 32646525

[cpp70246-bib-0108] Fowler, D. , and S. Morley . 1989. “The Cognitive‐Behavioural Treatment of Hallucinations and Delusions: A Preliminary Study.” Behavioural and Cognitive Psychotherapy 17, no. 3: 267–282. 10.1017/S0141347300016700.

[cpp70246-bib-0036] Fox, J. R. , N. S. Gray , and H. Lewis . 2004. “Factors Determining Compliance With Command Hallucinations With Violent Content: The Role of Social Rank, Perceived Power of the Voice and Voice Malevolence.” Journal of Forensic Psychiatry & Psychology 15, no. 3: 511–531. 10.1080/1478994042000226741.

[cpp70246-bib-0037] Georgiades, A. , A. Almuqrin , P. Rubinic , K. Mouhitzadeh , S. Tognin , and A. Mechelli . 2023. “Psychosocial Stress, Interpersonal Sensitivity, and Social Withdrawal in Clinical High Risk for Psychosis: A Systematic Review.” Schizophrenia (Heidelberg, Germany) 9, no. 1: 38. 10.1038/s41537-023-00362-z. 37330526 PMC10276848

[cpp70246-bib-0038] Ghadban, C. , S. Hallit , M. Achkar , et al. 2024. “Beliefs About Voices and Correlates in Long‐Stay Patients With Persistent Auditory Hallucinations, Diagnosed With Schizophrenia.” Psychosis 17, no. 3: 287–299. 10.1080/17522439.2024.2413527.

[cpp70246-bib-0039] Ghanem, M. , C. Evangeli‐Dawson , and A. Georgiades . 2023. “The Role of Culture on the Phenomenology of Hallucinations and Delusions, Explanatory Models, and Help‐Seeking Attitudes: A Narrative Review.” Early Intervention in Psychiatry 17, no. 9: 843–863. 10.1111/eip.13449. 37458202

[cpp70246-bib-0041] Gmeiner, A. , A. Gaglia , S. Habicher , et al. 2020. “Power to the Voice Hearer—The German Version of the Voice Power Differential Scale.” PLoS ONE 15, no. 3: e0230778. 10.1371/journal.pone.0230778. 32214352 PMC7098598

[cpp70246-bib-0042] Griffiths, S. L. , M. Michail , and M. Birchwood . 2012. “Cognitive Theory and Therapy for Command Hallucinations.” Journal of Experimental Psychopathology 3, no. 4: 537–551. 10.5127/jep.025811.

[cpp70246-bib-0043] Gurnani, R. , and A. Georgiades . 2025. “The Role of Emotion in Psychosis Onset and Symptom Persistence: A Systematic Review.” Early Intervention in Psychiatry 19, no. 10: e70096. 10.1111/eip.70096. 41045154 PMC12495843

[cpp70246-bib-0044] Hacker, D. , M. Birchwood , J. Tudway , A. Meaden , and C. Amphlett . 2008. “Acting on Voices: Omnipotence, Sources of Threat, and Safety‐Seeking Behaviours.” British Journal of Clinical Psychology 47, no. Pt 2: 201–213. 10.1348/014466507X249093. 17958943

[cpp70246-bib-0045] Haddock, G. , R. P. Bentall , and P. D. Slade . 1993. “Psychological Treatment of Chronic Auditory Hallucinations: Two Case Studies.” Behavioural and Cognitive Psychotherapy 21, no. 4: 335–346. 10.1017/S1352465800011668.

[cpp70246-bib-0120] Hartigan, N. , S. McCarthy‐Jones , and M. Hayward . 2014. “Hear Today, Not Gone Tomorrow? An Exploratory Longitudinal Study of Auditory Verbal Hallucinations (Hearing Voices).” Behavioural and Cognitive Psychotherapy 42: 117–123. 23866079 10.1017/S1352465813000611

[cpp70246-bib-0047] Hayward, M. 2003. “Interpersonal Relating and Voice Hearing: To What Extent Does Relating to the Voice Reflect Social Relating?” Psychology and Psychotherapy 76, no. Pt 4: 369–383. 10.1348/147608303770584737. 14670187

[cpp70246-bib-0048] Hayward, M. , R. Edgecumbe , A. M. Jones , C. Berry , and C. Strauss . 2018. “Brief Coping Strategy Enhancement for Distressing Voices: An Evaluation in Routine Clinical Practice.” Behavioural and Cognitive Psychotherapy 46, no. 2: 226–237. 10.1017/S1352465817000388. 28651663

[cpp70246-bib-0049] Hayward, M. , A. M. Jones , L. Bogen‐Johnston , N. Thomas , and C. Strauss . 2017. “Relating Therapy for Distressing Auditory Hallucinations: A Pilot Randomized Controlled Trial.” Schizophrenia Research 183: 137–142. 10.1016/j.schres.2016.11.019. 27916286

[cpp70246-bib-0050] Hazell, C. M. , S. Hasapopoulos , J. McGowan , et al. 2024. “The Role of Verbal Auditory Hallucinations in Influencing and Retrospectively Predicting Physical Harm Prevalence in Early Psychosis.” Clinical Practice and Epidemiology in Mental Health 20: e17450179286452. 10.2174/0117450179286452240520070533. 39130189 PMC11311800

[cpp70246-bib-0051] Hersh, K. , and R. Borum . 1998. “Command Hallucinations, Compliance, and Risk Assessment.” Journal of the American Academy of Psychiatry and the Law 26, no. 3: 353–359. 9785279

[cpp70246-bib-0052] Hustig, H. H. , and R. J. Hafner . 1990. “Persistent Auditory Hallucinations and Their Relationship to Delusions and Mood.” Journal of Nervous and Mental Disease 178, no. 4: 264–267. 10.1097/00005053-199004000-00009. 2319235

[cpp70246-bib-0053] Jorovat, A. , R. Twumasi , A. Mechelli , and A. Georgiades . 2025. “Core Beliefs in Psychosis: A Systematic Review and Meta‐Analysis.” Schizophrenia (Heidelberg, Germany) 11, no. 1: 38. 10.1038/s41537-025-00577-2. 40050627 PMC11885481

[cpp70246-bib-0054] Junginger, J. 1990. “Predicting Compliance With Command Hallucinations.” American Journal of Psychiatry 147, no. 2: 245–247. 10.1176/ajp.147.2.245. 2301669

[cpp70246-bib-0055] Junginger, J. 1995. “Command Hallucinations and the Prediction of Dangerousness.” Psychiatric Services (Washington, D.C.) 46, no. 9: 911–914. 10.1176/ps.46.9.911. 7583501

[cpp70246-bib-0056] Kalhovde, A. M. , I. Elstad , and A. G. Talseth . 2014. ““Sometimes I Walk and Walk, Hoping to Get Some Peace.” Dealing With Hearing Voices and Sounds Nobody Else Hears.” International Journal of Qualitative Studies on Health and Well‐Being 9: 23069. 10.3402/qhw.v9.23069. 24674764 PMC3968296

[cpp70246-bib-0109] Kingdon, D. G. , and D. Turkington . 1991. “The Use of Cognitive Behavior Therapy with a Normalizing Rationale in Schizophrenia. Preliminary Report.” Journal of Nervous and Mental Disease 179, no. 4: 207–211. 10.1097/00005053-199104000-00005. 2007891

[cpp70246-bib-0057] Larøi, F. , N. Thomas , A. Aleman , et al. 2019. “The Ice in Voices: Understanding Negative Content in Auditory‐Verbal Hallucinations.” Clinical Psychology Review 67: 1–10. 10.1016/j.cpr.2018.11.001. 30553563

[cpp70246-bib-0058] Lee, T. M. , S. A. Chong , Y. H. Chan , and G. Sathyadevan . 2004. “Command Hallucinations Among Asian Patients With Schizophrenia.” Canadian Journal of Psychiatry. Revue Canadienne de Psychiatrie 49, no. 12: 838–842. 10.1177/070674370404901207. 15679207

[cpp70246-bib-0121] Lincoln, T. M. , and E. Peters . 2019. “A Systematic Review and Discussion of Symptom Specific Cognitive Behavioural Approaches to Delusions and Hallucinations.” Schizophrenia Research 203: 66–79. 10.1016/j.schres.2017.12.014. 29352708

[cpp70246-bib-0059] Lockwood, C. , Z. Munn , and K. Porritt . 2015. “Qualitative Research Synthesis: Methodological Guidance for Systematic Reviewers Utilizing Metaaggregation.” International Journal of Evidence‐Based Healthcare 13, no. 3: 179–187. 26262565 10.1097/XEB.0000000000000062

[cpp70246-bib-0061] Lucas, S. , and T. Wade . 2001. “An Examination of the Power of the Voices in Predicting the Mental State of People Experiencing Psychosis.” Behaviour Change 18, no. 1: 51–57. 10.1375/bech.18.1.51.

[cpp70246-bib-0062] Luhrmann, T. M. , R. Padmavati , H. Tharoor , and A. Osei . 2015. “Differences in Voice‐Hearing Experiences of People With Psychosis in the U.S.A., India and Ghana: Interview‐Based Study.” British Journal of Psychiatry: The Journal of Mental Science 206, no. 1: 41–44. 10.1192/bjp.bp.113.139048. 24970772

[cpp70246-bib-0063] Mackinnon, A. , D. L. Copolov , and T. Trauer . 2004. “Factors Associated With Compliance and Resistance to Command Hallucinations.” Journal of Nervous and Mental Disease 192, no. 5: 357–362. 10.1097/01.nmd.0000126728.70060.fa. 15126890

[cpp70246-bib-0064] Marotti, J. , R. Saunders , A. Montague , and M. Fornells‐Ambrojo . 2025. “The Role of Trauma, Attachment, and Voice‐Hearer's Appraisals: A Latent Profile Analysis in the AVATAR2 Trial.” Psychological Medicine 55: e65. 10.1017/S003329172500008X. 40012531 PMC12080651

[cpp70246-bib-0107] Meaden, A. , N. Keen , R. Aston , K. Barton , and S. Bucci . 2013. Cognitive Therapy for Command Hallucinations: An Advanced Practical Companion. Routledge.

[cpp70246-bib-0065] Morris, E. M. , P. Garety , and E. Peters . 2014. “Psychological Flexibility and Nonjudgemental Acceptance in Voice Hearers: Relationships With Omnipotence and Distress.” Australian and New Zealand Journal of Psychiatry 48, no. 12: 1150–1162. 10.1177/0004867414535671. 24835207

[cpp70246-bib-0068] Murphy, A. , E. Taylor , and R. Elliott . 2012. “The Detrimental Effects of Emotional Process Dys Regulation on Decision‐Making in Substance Dependence.” Frontiers in Integrative Neuroscience 6: 101. 23162443 10.3389/fnint.2012.00101 PMC3491319

[cpp70246-bib-0105] Page, M. J. , J. E. McKenzie , P. M. Bossuyt , et al. 2021. “The PRISMA 2020 Statement: An Updated Guideline for Reporting Systematic Reviews.” BMJ 372: n71. 10.1136/bmj.n71. 33782057 PMC8005924

[cpp70246-bib-0122] Paulik, G. 2012. “The Role of Social Schema in the Experience of Auditory Hallucinations: A Systematic Review and a Proposal for the Inclusion of Social Schema in a Cognitive Behavioural Model of Voice Hearing.” Clinical Psychology & Psychotherapy 19, no. 6: 459–472. 10.1002/cpp.768. 21774037

[cpp70246-bib-0072] Peters, E. R. , S. L. Williams , M. A. Cooke , and E. Kuipers . 2012. “It's Not What You Hear, It's the Way You Think About It: Appraisals as Determinants of Affect and Behaviour in Voice Hearers.” Psychological Medicine 42, no. 7: 1507–1514. 10.1017/S0033291711002650. 22115329

[cpp70246-bib-0073] Pontillo, M. , F. De Crescenzo , S. Vicari , et al. 2016. “Cognitive Behavioural Therapy for Auditory Hallucinations in Schizophrenia: A Review.” World Journal of Psychiatry 6, no. 3: 372–380. 10.5498/wjp.v6.i3.372. 27679778 PMC5031939

[cpp70246-bib-0074] Rajanthiran, L. , G. Curtis , J. Ayalde , et al. 2022. “‘Is Hearing Believing?’: A Study of Voices and Beliefs in Psychosis and Trauma.” Australasian Psychiatry: Bulletin of Royal Australian and New Zealand College of Psychiatrists 30, no. 4: 547–551. 10.1177/10398562221106064. 35968743

[cpp70246-bib-0110] Read, J. , P. Hammersley , and T. Rudegeair . 2007. “Why, When and How to Ask About Childhood Abuse.” Advances in Psychiatric Treatment 13, no. 2: 101–110.

[cpp70246-bib-0075] Reynolds, N. , and P. Scragg . 2010. “Compliance With Command Hallucinations: The Role of Power in Relation to the Voice, and Social Rank in Relation to the Voice and Others.” Journal of Forensic Psychiatry & Psychology 21, no. 1: 121–138. 10.1080/14789940903194111.

[cpp70246-bib-0076] Robles‐García, R. , F. Páez Agraz , O. Zúñiga Partida , A. Rizo Méndez , and E. Hernández Villanueva . 2004. “Estudio de Traducción al Español y Propiedades Psicométricas del Cuestionario de Creencias Acerca de las Voces (BAVQ) [Beliefs About Voices Questionnaire (BAVQ): Spanish Translation and Psychometric Properties].” Actas Españolas de Psiquiatría 32, no. 6: 358–362. 15529225

[cpp70246-bib-0077] Robson, G. , and O. Mason . 2015. “Interpersonal Processes and Attachment in Voice‐Hearers.” Behavioural and Cognitive Psychotherapy 43, no. 6: 655–668. 10.1017/S1352465814000125. 24780442

[cpp70246-bib-0078] Rogers, P. , A. Watt , N. S. Gray , M. MacCulloch , and K. Gournay . 2002. “Content of Command Hallucinations Predicts Self‐Harm but Not Violence in a Medium Secure Unit.” Journal of Forensic Psychiatry 13, no. 2: 251–262. 10.1080/09585180210150096.

[cpp70246-bib-0115] Rudnick, A. 1999. “Command Hallucinations and Dangerous Behaviour.” Journal of the American Academy of Psychiatry and Law 27: 253–257. 10400433

[cpp70246-bib-0080] Salim, Z. , C. Haddad , S. Obeid , E. Awad , S. Hallit , and G. Haddad . 2021. “Command Voices and Aggression in a Lebanese Sample Patients With Schizophrenia.” Psychiatria Danubina 33, no. 1: 27–35. 10.24869/psyd.2021.27. 33857037

[cpp70246-bib-0081] Sandelowski, M. , C. I. Voils , and J. Barroso . 2006. “Defining and Designing Mixed Research Synthesis Studies.” Research in the Schools: A Nationally Refereed Journal Sponsored by the Mid‐South Educational Research Association and the University of Alabama 13, no. 1: 29. 20098638 PMC2809982

[cpp70246-bib-0082] Sayer, J. , S. Ritter , and K. Gournay . 2000. “Beliefs About Voices and Their Effects on Coping Strategies.” Journal of Advanced Nursing 31, no. 5: 1199–1205. 10.1046/j.1365-2648.2000.01375.x. 10840254

[cpp70246-bib-0083] Shawyer, F. , J. Farhall , A. Mackinnon , et al. 2012. “A Randomised Controlled Trial of Acceptance‐Based Cognitive Behavioural Therapy for Command Hallucinations in Psychotic Disorders.” Behaviour Research and Therapy 50, no. 2: 110–121. 10.1016/j.brat.2011.11.007. 22186135

[cpp70246-bib-0084] Shawyer, F. , A. Mackinnon , J. Farhall , et al. 2008. “Acting on Harmful Command Hallucinations in Psychotic Disorders: An Integrative Approach.” Journal of Nervous and Mental Disease 196, no. 5: 390–398. 10.1097/NMD.0b013e318171093b. 18477881

[cpp70246-bib-0085] Shawyer, F. , A. Mackinnon , J. Farhall , T. Trauer , and D. Copolov . 2003. “Command Hallucinations and Violence: Implications for Detention and Treatment.” Psychiatry, Psychology and Law 10, no. 1: 97–107. 10.1375/pplt.2003.10.1.97.

[cpp70246-bib-0086] Shawyer, F. , K. Ratcliff , A. Mackinnon , J. Farhall , S. C. Hayes , and D. Copolov . 2007. “The Voices Acceptance and Action Scale (VAAS): Pilot Data.” Journal of Clinical Psychology 63, no. 6: 593–606. 10.1002/jclp.20366. 17457846

[cpp70246-bib-0112] Sheitman, B. B. , B. J. Kinon , B. A. Ridgway , and J. A. Lieberman . 1998. “Pharmacological Treatments of Schizophrenia.” In A Guide to Treatments That Work, edited by P. E. Nathan and J. M. Gorman , 167–189. Oxford University Press.

[cpp70246-bib-0087] Simms, J. , V. McCormack , R. Anderson , and C. Mulholland . 2007. “Correlates of Self‐Harm Behaviour in Acutely Ill Patients With Schizophrenia.” Psychology and Psychotherapy 80, no. Pt 1: 39–49. 10.1348/147608306X99386. 17346379

[cpp70246-bib-0117] Smailes, D. , B. Alderson‐Day , C. Fernyhough , S. McCarthy‐Jones , and G. Dodgson . 2015. “Tailoring Cognitive Behavioral Therapy to Subtypes of Voice‐Hearing.” Frontiers in Psychology 6: 1933. 10.3389/fpsyg.2015.01933. 26733919 PMC4685120

[cpp70246-bib-0089] So, S. H. , M. J. Begemann , X. Gong , and I. E. Sommer . 2016. “Relationship Between Neuroticism, Childhood Trauma and Cognitive‐Affective Responses to Auditory Verbal Hallucinations.” Scientific Reports 6: 34401. 10.1038/srep34401. 27698407 PMC5048145

[cpp70246-bib-0090] So, S. H. , L. K. Chung , C. Y. Tse , et al. 2021. “Moment‐To‐Moment Dynamics Between Auditory Verbal Hallucinations and Negative Affect and the Role of Beliefs About Voices.” Psychological Medicine 51, no. 4: 661–667. 10.1017/S0033291719003611. 31907105

[cpp70246-bib-0091] So, S. H. , and C. W. Wong . 2008. “Experience and Coping With Auditory Hallucinations in First‐Episode Psychosis: Relationship With Stress Coping.” Hong Kong Journal of Psychiatry 18, no. 3: 115–122.

[cpp70246-bib-0092] Soppitt, R. W. , and M. Birchwood . 1997. “Depression, Beliefs, Voice Content and Topography: A Cross‐sectional Study of Schizophrenic Patients with Auditory Verbal Hallucinations.” Journal of Mental Health 6, no. 5: 525–532. 10.1080/09638239718617.

[cpp70246-bib-0093] Stephanie, L. , R. Susan L , T. Wei Lin , S. Monique , and T. Neil . 2018. “Does Mindfulness Help People Adapt to the Experience of Hearing Voices?” Psychiatry Research 270: 329–334. 10.1016/j.psychres.2018.09.013. 30292085

[cpp70246-bib-0094] Strauss, C. , K. Hugdahl , F. Waters , et al. 2018. “The Beliefs About Voices Questionnaire–Revised: A Factor Structure From 450 Participants.” Psychiatry Research 259: 95–103. 10.1016/j.psychres.2017. 29035759 PMC5764292

[cpp70246-bib-0095] Suryani, S. , A. Welch , and L. Cox . 2013. “The Phenomena of Auditory Hallucination as Described by Indonesian People Living With Schizophrenia.” Archives of Psychiatric Nursing 27, no. 6: 312–318. 10.1016/j.apnu.2013.08.001. 24238012

[cpp70246-bib-0096] Thomas, H. 2003. “Quality Assessment Tool for Quantitative Studies. Effective Public Health Practice Project.” http://www.ephpp.ca/PDF/Quality%20Assessment%20Tool_2010_2.pdf.

[cpp70246-bib-0097] Trower, P. , M. Birchwood , A. Meaden , S. Byrne , A. Nelson , and K. Ross . 2004. “Cognitive Therapy for Command Hallucinations: Randomised Controlled Trial.” British Journal of Psychiatry: The Journal of Mental Science 184: 312–320. 10.1192/bjp.184.4.312. 15056575

[cpp70246-bib-0099] van der Gaag, M. , M. C. Hageman , and M. Birchwood . 2003. “Evidence for a Cognitive Model of Auditory Hallucinations.” Journal of Nervous and Mental Disease 191, no. 8: 542–545. 10.1097/01.nmd.0000082183.95853.ec. 12972858

[cpp70246-bib-0101] Westhead, M. , and A. Georgiades . 2025. “The Role of Spirituality and Religiosity in the Maintenance and Recovery of Psychosis: A Systematic Review.” Early Intervention in Psychiatry 19, no. 7: e70061. 10.1111/eip.70061. 40605616 PMC12223707

[cpp70246-bib-0102] Wong, Z. , D. Öngür , B. Cohen , C. Ravichandran , G. Noam , and B. Murphy . 2013. “Command Hallucinations and Clinical Characteristics of Suicidality in Patients With Psychotic Spectrum Disorders.” Comprehensive Psychiatry 54, no. 6: 611–617. 10.1016/j.comppsych.2012.12.022. 23375263

[cpp70246-bib-0103] World Health Organization . 2019. International Statistical Classification of Diseases and Related Health Problems, 11th ed. World Health Organization. https://icd.who.int/.

[cpp70246-bib-0104] Zanello, A. , and J. R. Dugré . 2021. “Preliminary Evidence for Heterogeneity of Beliefs About Auditory Verbal Hallucinations Intent.” Journal of Nervous and Mental Disease 209, no. 12: 872–878. 10.1097/NMD.0000000000001391. 34846355

